# KARR-seq reveals cellular higher-order RNA structures and RNA–RNA interactions

**DOI:** 10.1038/s41587-023-02109-8

**Published:** 2024-01-18

**Authors:** Tong Wu, Anthony Youzhi Cheng, Yuexiu Zhang, Jiayu Xu, Jinjun Wu, Li Wen, Xiao Li, Bei Liu, Xiaoyang Dou, Pingluan Wang, Linda Zhang, Jingyi Fei, Jianrong Li, Zhengqing Ouyang, Chuan He

**Affiliations:** 1 Department of Chemistry, University of Chicago, Chicago, IL USA; 2 Howard Hughes Medical Institute, Chicago, IL USA; 3 Department of Genetics and Genome Sciences and Institute for Systems Genomics, University of Connecticut, Farmington, CT USA; 4 Department of Biostatistics and Epidemiology, School of Public Health and Health Sciences, University of Massachusetts, Amherst, MA USA; 5 Department of Veterinary Biosciences, College of Veterinary Medicine, The Ohio State University, Columbus, OH USA; 6 Department of Biochemistry and Molecular Biology, Institute for Biophysical Dynamics, University of Chicago, Chicago, IL USA; 7 Present Address: Genome Institute of Singapore, Agency for Science, Technology and Research (A*STAR), Singapore, Singapore

**Keywords:** RNA metabolism, RNA sequencing, RNA

## Abstract

RNA fate and function are affected by their structures and interactomes. However, how RNA and RNA-binding proteins (RBPs) assemble into higher-order structures and how RNA molecules may interact with each other to facilitate functions remain largely unknown. Here we present KARR-seq, which uses N_3_-kethoxal labeling and multifunctional chemical crosslinkers to covalently trap and determine RNA–RNA interactions and higher-order RNA structures inside cells, independent of local protein binding to RNA. KARR-seq depicts higher-order RNA structure and detects widespread intermolecular RNA–RNA interactions with high sensitivity and accuracy. Using KARR-seq, we show that translation represses mRNA compaction under native and stress conditions. We determined the higher-order RNA structures of respiratory syncytial virus (RSV) and vesicular stomatitis virus (VSV) and identified RNA–RNA interactions between the viruses and the host RNAs that potentially regulate viral replication.

## Abstract (web summary)

RNA interactions and structures are determined with a chemical labeling and crosslinking method.

## Main

RNA lies in the center of gene expression regulation primarily through its interactions with other biomacromolecules, such as RNA-binding proteins (RBPs), DNA and other RNA species. Pioneered by approaches involving crosslinking and immunoprecipitation (CLIP)^1^, various methods have been developed to study RNA–RBP interactions, which have markedly advanced RNA biology. However, how RNAs interact with other molecules and the subsequent functional consequences remain inadequately studied. The functions of RNAs are usually determined by their higher-order structures^2^, which include the assembly and organization of multiple RNA secondary structural elements as well as three-dimensional (3D) RNA conformations mediated by other biomacromolecules. However, these structures are difficult to determine due to their highly dynamic nature, in particular when responding to perturbations.

High-throughput sequencing-based approaches have been applied to reveal RNA–RNA interactions transcriptome wide. Intramolecular RNA–RNA interactions are often critical to the functional relevance of higher-order RNA structures, and intermolecular interactions may reflect distinct RNA functions. Psoralen-based methods, such as PARIS, RAP-RNA, LIGR-seq, SPLASH and COMRADES^3–7^, directly capture RNA duplexes and were applied to study the impact of pairwise RNA interactions on RNA metabolism. These methods predominantly capture base-pairing interactions but may miss the distance information between spatially proximal single-stranded RNA fragments. Protein-mediated approaches, including CLASH, RPL, MARIO and RIPPLiT^8–11^, complement psoralen crosslinking by revealing physical distances between RNAs, but they usually exhibit limited sensitivity for transcripts with modest expression levels and mostly capture RNAs bound by particular proteins. RIC-seq improved the sensitivity by performing protein-mediated proximity ligation in cells and incorporating biotinylated nucleotides during the ligation step^12^. However, because the local protein concentration and the strength of protein–RNA association are spatially heterogeneous^13^, RNA regions with weak protein–RNA engagement could be underrepresented. Therefore, it is desirable to develop technologies that do not rely solely on protein–RNA crosslinking to study higher-order RNA structures and RNA–RNA interactions.

Here we describe kethoxal-assisted RNA–RNA interaction sequencing (KARR-seq), which takes advantage of N_3_-kethoxal-mediated RNA labeling^14^ and dendrimer-based nucleic acid conformation capture^15^. N_3_-kethoxal has been demonstrated to efficiently label RNA with azide groups in live cells, serving as a bio-orthogonal handle for further functionalization^14^. Meanwhile, PAMAM dendrimers modified by multiple dibenzocyclooctane (DBCO) moieties can readily crosslink proximal RNA transcripts by reacting with the labeled azide groups. Because KARR-seq uses chemical crosslinkers to capture spatially proximal transcripts, RNA–RNA interactions detected by KARR-seq are not determined solely by local protein concentrations or RBP–RNA affinities. The size of crosslinkers can be tuned to capture proximity at different distances. KARR-seq also enables the enrichment of crosslinked products, increasing the sensitivity for transcripts with relatively low abundance.

KARR-seq accurately reveals RNA tertiary structures and identifies intermolecular RNA–RNA interactions. Using KARR-seq, we show that cytoplasmic mRNA has less compact structures than their nuclear counterparts, with translation resolving higher-order RNA structures under native and stress conditions. We detected RNA–RNA interactions that affect pre-rRNA processing. Furthermore, we mapped the tertiary structures of respiratory syncytial virus (RSV) and vesicular stomatitis virus (VSV) RNAs and detected hundreds of interactions between viral and host RNAs. Host mRNAs that interact with RSV and VSV enrich different molecular pathways. The blockage of RNA–RNA interactions between RSV RNA and host mRNAs represses RSV replication. KARR-seq thus enables precise and sensitive mapping of RNA–RNA interactions and RNA structurome to reveal RNA functions.

## Results

### Development of KARR-seq

Effective capture of spatially proximal RNAs has been challenging primarily owing to modest RNA crosslinking efficiency by limited available RNA crosslinkers^16^. Click chemistry reactions happen fast and quantitatively in cells under ambient conditions, enabling the detection of binding landscapes of many biomacromolecules^17^. However, these reactions have not been applied in RNA proximity studies due to a lack of ‘clickable’ functional groups on cellular RNA. In KARR-seq, we applied N_3_-kethoxal, a cell-permeable and nucleus-permeable small molecule that efficiently functionalizes RNA with an azide tag^14,18^. We also decorated commercially available dendrimers with multiple DBCO and biotin moieties (Supplementary Fig. 1a,b), with DBCO reacting with proximal N_3_-kethoxal-modified RNA via ‘click’ reactions and biotin enabling enrichment of the crosslinked products.

**Fig. 1:**
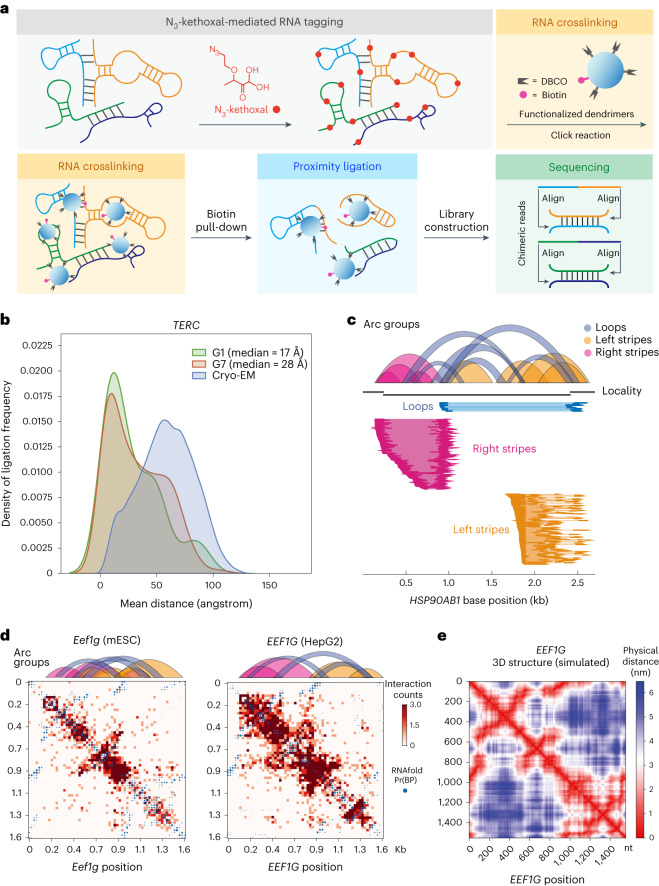
KARR-seq maps higher-order RNA structures. **a**, KARR-seq workflow. Cells are first treated with N_3_-kethoxal to modify RNAs with azide tags (red), which enables crosslinking of the tagged RNA molecules by DBCO-decorated dendrimers (blue). Biotin modifications (pink) on dendrimers facilitate the enrichment of crosslinking products, followed by proximity ligation, RNA library construction and sequencing. Chimeric sequencing reads are aligned to identify RNA–RNA interactions. **b**, Physical distances between interacting fragments of *TERC* in K562 cells, measured by KARR-seq data generated using G1 and G7 dendrimers, respectively. The physical distances were measured using the cryo-EM structure of *TERC*. The actual physical distance distribution in the cryo-EM structure is shown in blue for comparison. **c**, Illustration of loop and stripe structures detected by KARR-seq. In arc groups, loops, left stripes and right stripes are denoted in blue, yellow and pink, respectively. Corresponding KARR-seq chimeric reads are displayed below. **d**, The KARR-seq interaction maps and arc groups for the *Eef1g* (*EEF1G*) transcript in mESCs (left) and HepG2 cells (right). **e**, The simulated physical distance map of the human *EEF1G* transcript. For **b**–**d**, KARR-seq was performed in two biological replicates.

We first labeled fixed and permeabilized murine embryonic stem cells (mESCs) with N_3_-kethoxal and then diffuse modified dendrimer G3 at 37 °C to initiate the ‘click’ reaction (Fig. 1a). Gel electrophoresis (Supplementary Fig. 1c) and dot blot (Supplementary Fig. 1d) of the purified RNA confirmed successful RNA crosslinking. Control experiments performed in the absence of N_3_-kethoxal or dendrimer showed very weak or invisible signals in dot blot (Supplementary Fig. 1d). RNA from crosslinked cells was then fragmented and applied for pull-down using streptavidin-coated beads. On-bead end repair and proximity ligation were subsequently performed, and the post-ligation RNA was amplified for pair-ended sequencing for roughly 100 million reads per sample (Fig. 1a).

De-duplicated KARR-seq chimeric reads constituted around 6% of all reads and recapitulate ribosomal RNA (rRNA) higher-order structures (Supplementary Fig. 1e). In negative controls performed in the absence of N_3_-kethoxal, chimeric reads constitute only 0.26% of sequencing reads. RNA contact maps generated from individual KARR-seq replicates show high correlations (Supplementary Fig. 1f). Because N_3_-kethoxal specifically reacts with guanines^14^, we evaluated the effect of nucleotide content on the frequency of KARR-seq chimeric reads. Using data produced by G1 dendrimers, we analyzed transcripts with more than 250 chimeric reads and grouped all base positions according to their chimeric reads coverage. We found that guanine is modestly enriched among bases with high chimeric reads coverage (Supplementary Fig. 1g). We then performed KARR-seq with a 1:1 mixture of human (K562) and *Drosophila* (S2) cells. In this case, chimeric reads constitute 8.4% of total reads when mapped to the reference genomes. Interspecies chimeric reads account for 7.2% of chimeric reads (Supplementary Fig. 2a) and 0.61% of all sequencing reads, with no significant interaction detected between human and *Drosophila* RNAs (Supplementary Fig. 2b). The percentage of interspecies chimeric reads for KARR-seq (0.61%) is similar to that in RIC-seq (0.6%)^12^. Note that, in RIC-seq, the proximity ligation reaction was performed in fixed cells instead of free solutions, and, therefore, the interspecies ligation frequency is expected to be low.

We next analyzed KARR-seq data produced by dendrimers with different diameters, namely G1 (22 Å), G3 (36 Å), G5 (54 Å) and G7 (81 Å), in mESCs and K562 cells. We projected KARR-seq chimeric read positions onto the 3D cryogenic electron microscopy (cryo-EM) structures of *TERC* and U1 and calculated the spatial distances between the two RNA fragments from each chimeric read. For both transcripts, G7 captures a larger median distance than G1 does (Fig. 1b and Supplementary Fig. 3a). Dendrimers with similar sizes detect similar transcriptome-wide RNA–RNA interaction landscapes (Supplementary Fig. 3b,c). The choice of dendrimers did not affect ligation efficiency (Supplementary Fig. 3d), but G1 and G3 captured twice the amount of transcripts with valid interactions as G7 did (Supplementary Fig. 3e), potentially because G1 and G3 are more accessible to compact ribonucleoprotein (RNP) complexes^19^. We, therefore, used G1 for KARR-seq experiments unless otherwise noted.

### KARR-seq maps higher-order RNA structures

KARR-seq chimeric reads reveal both intra-molecular and inter-molecular RNA–RNA interactions (Supplementary Fig. 3c). We first evaluated the behavior of KARR-seq in depicting higher-order RNA structures in HepG2 cells, K562 cells and mESCs. We plotted the KARR-seq interaction map of human 18S rRNA from K562 cells and found that KARR-seq contact frequency recapitulates main features of the 18S rRNA physical distances map revealed by cryo-EM^20^ (Supplementary Fig. 4a). The distribution of physical distance revealed by KARR-seq overlaps decently with the actual distribution revealed by cryo-EM (Supplementary Fig. 4b). In comparison, RIC-seq and PARIS enrich interactions within short physical distances (Supplementary Fig. 4b), suggesting that KARR-seq may capture RNA proximity in a broader spatial distance range.

Compared to rRNA, mapping mRNA tertiary structures is particularly challenging owing to their dynamic nature and the relatively low abundance of mRNAs. KARR-seq detects mRNA loops and stripes, where loops stand for relatively stable duplex interactions and stripes represent more dynamic contacts or RNA–RNA proximity without direct pairing (Fig. 1c and Methods). We simulated RNA tertiary structures based on the freely jointed chain (FJC) model. Benchmarking with the cryo-EM structure using *RPPH1* resulted in a Pearson’s correlation coefficient of 0.696 between the actual and simulated physical distance maps (Supplementary Fig. 4c), indicating decent accuracy of the simulation. KARR-seq interaction frequency maps of mRNAs match the corresponding simulated physical distance maps (Fig. 1d,e), demonstrating the capability of KARR-seq in mRNA tertiary structure depiction. Interaction maps for homologous transcripts in HepG2 cells and mESCs reveal similar topologies (Fig. 1d), suggesting conserved RNA tertiary structure in different species.

### Benchmarking KARR-seq with RIC-seq and PARIS

**Fig. 2:**
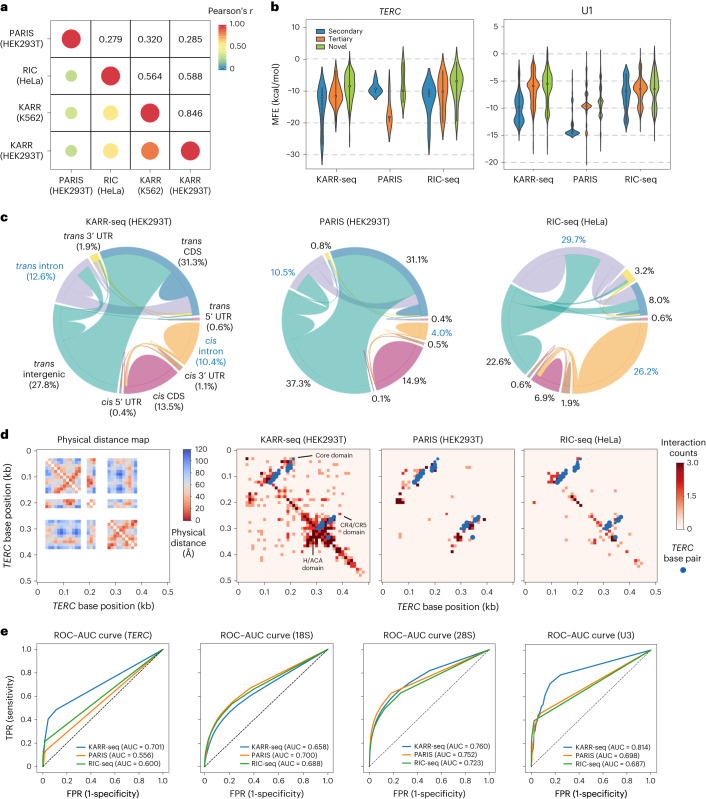
Benchmarking KARR-seq, RIC-seq and PARIS. **a**, Average Pearson correlation between the interaction maps of KARR-seq (K562 and HEK293T cells), RIC-seq (HeLa cells) and PARIS (HEK293T cells). **b**, MFE for RNA–RNA interactions detected using KARR-seq, PARIS and RIC-seq within *TERC* and U1 transcripts, respectively. Interactions were grouped into ‘secondary’, ‘tertiary’ and ‘novel’. ‘Secondary’ refers to the interactions that match secondary structure prediction. ‘Tertiary’ refers to spatially proximal RNA regions revealed by the cryo-EM structure that do not correspond to secondary structures. ‘Novel’ refers to interactions that are not supported by secondary structures or cryo-EM structures. **c**, Circos plots showing the RNA–RNA interaction landscape revealed by KARR-seq, PARIS and RIC-seq. The width of the link between two RNA categories indicates the relative abundance of chimeric reads taken by interactions between these two categories. **d**, Left, the physical distance map of *TERC* revealed by the cryo-EM structure of *TERC*. Right, higher-order structures of *TERC* detected by KARR-seq, PARIS and RIC-seq under the same sequencing depth. The blue dots denote base-pairing secondary structures acquired from the Rfam annotations (RF00024). **e**, The ROC–AUC curves for KARR-seq, RIC-seq and PARIS for detecting higher-order structures of *TERC*, 18S, 28S and U3. The dashed lines denote random classifiers. RIC-seq and PARIS data were acquired from the Gene Expression Omnibus (RIC-seq: GSE127188; PARIS: GSE74353). Cryo-EM structures were acquired from the Protein Data Bank (accession codes: 7QXB for *TERC*, 6QX9 for U3 and 4V6X for 18S and 28S). KARR-seq was performed in two biological replicates.

We systematically compared KARR-seq data (HEK293T, K562 and HepG2 cells) and results from PARIS (HEK293T cells)^4^ and RIC-seq (HeLa cells)^12^; PARIS and RIC-seq represent the state-of-the-art methods in mapping RNA duplexes and protein-mediated RNA proximity, respectively. KARR-seq exhibits a stronger correlation with RIC-seq than with PARIS (Fig. 2a), because both KARR-seq and RIC-seq detect spatially proximal RNA, whereas psoralen primarily reacts with duplexes. We next analyzed the minimal free energy (MFE) of the intramolecular RNA–RNA interactions detected by KARR-seq, PARIS and RIC-seq on *TERC* and U1 and divided all interactions into three categories depending on whether they match with cryo-EM structures. Interactions detected by PARIS mostly correspond to known secondary structures and tend to have low mean MFE, whereas interactions detected by KARR-seq and RIC-seq include more spatially proximal non-duplex contacts (denoted as tertiary; Fig. 2b) and interactions that are not revealed by cryo-EM (denoted as novel; Fig. 2b). All three methods share a similar ratio of chimeric reads (5–7%) when data were mapped to the human genome (Supplementary Fig. 5a). However, the percentage of chimeric reads dropped to only around 1% when RIC-seq data were mapped to the transcriptome (Supplementary Fig. 5a), indicating that RIC-seq chimeric reads enrich pre-mRNA. Transcripts detected by RIC-seq exhibit lower expression levels (Supplementary Fig. 5b) and are longer (Supplementary Fig. 5c) than those detected by KARR-seq and PARIS. Concordantly, KARR-seq and PARIS share a similar RNA–RNA interaction landscape between different RNA categories, whereas the majority (56%) of interactions identified by RIC-seq are intron mediated (Fig. 2d), further suggesting that RIC-seq enriches interactions in the cell nucleus.

We then performed differential analysis of RNA–RNA interactions detected by KARR-seq and RIC-seq. Interactions uniquely detected by KARR-seq are mostly stripes, whereas RIC-seq-specific interactions are mostly loops (Supplementary Fig. 5d), suggesting that KARR-seq could detect more transient and dynamic contacts. Around half of KARR-seq-specific intramolecular interactions on mRNAs are located at coding sequences (CDS), whereas 84% of RIC-seq-specific intramolecular interactions on mRNAs are at the 3′ untranslated region (UTR) (Supplementary Fig. 5e), which could be due to the binding preference of specific RBPs^21^.

We next benchmarked KARR-seq, PARIS and RIC-seq using transcripts with published cryo-EM structures, including 18S, 28S, U3 and *TERC*. KARR-seq detects pervasive intramolecular RNA–RNA interactions and recapitulates the physical distance maps (Fig. 2d and Supplementary Fig. 6a). Quantitatively, receiver operating characteristic (ROC) analysis revealed higher or similar area under the curve (AUC) numbers of KARR-seq than the other two methods (Fig. 2e). Note that KARR-seq, PARIS and RIC-seq datasets were generated from different cell lines and were sequenced to different depths, which could complicate direct comparisons.

Because psoralen crosslinks double-stranded RNA, PARIS data show segment-like patterns that represent RNA duplexes on the contact maps. In comparison, kethoxal predominantly reacts with single-stranded regions. Therefore, KARR-seq data present triangular domain-like structures (Fig. 2d and Supplementary Fig. 6b,c). Notably, ‘KARR-seq domains’ cover similar nucleotide regions as ‘PARIS segments’ do (Fig. 2d and Supplementary Fig. 6b,c), suggesting that PARIS and KARR-seq reveal different possible RNA conformations at the same RNA region to complement each other.

### Distinct mRNA higher-order structures in the cell nucleus

RNA secondary structures are dynamic when RNAs transit from the nucleus to cytoplasm^22^. However, higher-order RNA structures among different cellular compartments have not been characterized. We purified K562 nuclei (Supplementary Fig. 7a) for KARR-seq, with dot blot showing a comparable RNA-labeling efficiency in purified nuclei and intact K562 cells (Supplementary Fig. 7b). KARR-seq data from the nuclear fraction differ evidently from that using intact cells (Supplementary Fig. 7c). Transcripts with valid higher-order structures detected in the nucleus are longer and show lower expression levels than those detected in intact cells (Supplementary Fig. 7d,e). Notably, transcripts detected by KARR-seq using cell nuclei and transcripts detected by RIC-seq using intact cells display similar length and expression level (Supplementary Fig. 7d,e). These results suggest distinct higher-order RNA structure landscapes between the nucleus and cytoplasm and corroborate that RIC-seq enriches nuclear RNA–RNA interactions.

We further investigated the higher-order structure differences between nuclear and cytoplasmic RNA in K562 cells. We also performed KARR-seq in vitro using purified and refolded K562 total RNA, which reveals the intrinsic ability of RNA polynucleotide chain to fold in the absence of cellular factors. RNA contact frequency decreases log-linearly to the coordinate distance in all tested conditions. The slope of the trend lines, defined as beta coefficients (*β*), is −2.06 and −1.63 for mRNA in intact cells and the nuclei, respectively, with more long-range contacts detected in the nuclear fraction (Supplementary Fig. 8a). We calculated the beta coefficient for each individual transcript. RNAs within the nuclei tend to have higher beta coefficient compared to same transcripts from intact cells, shown as a transcriptome-wide distribution (Supplementary Fig. 8b) or scrutinized at the level of individual transcripts (Supplementary Fig. 8c–e).

In the KARR-seq protocol, cells are pre-fixed using 1% formaldehyde before N_3_-kethoxal treatment. We assayed the effect of formaldehyde crosslinking by performing KARR-seq using weakly fixed (0.1% formaldehyde) and unfixed K562 cells. Under 1% and 0.1% formaldehyde conditions, RNA–RNA interactions detected by KARR-seq are largely similar both at the transcript level (Supplementary Fig. 9a,b) and transcriptome wide (Supplementary Fig. 9c,d). However, interaction maps generated from the unfixed cells resemble those for refolded RNA (Supplementary Fig. 9a,b). Quantitatively, we observed higher beta coefficients across the transcriptome along with more long-range interactions using unfixed cells (Supplementary Fig. 9c,d). We speculate that weakly bound RBPs and ribosomes could fall off from RNA during the labeling steps in the absence of formaldehyde, which could lead to partial RNA refolding. Formaldehyde crosslinking is likely necessary to capture bona fide cellular RNA conformations using KARR-seq.

To quantify the extent of RNA folding across transcripts with varying lengths and abundance, we devised the folding index, an exponential decay transformation of the genomic distance between the two arms of each chimeric read^23^ (Methods). The folding index describes the relative genomic distance of two interacting RNA fragments, with a high folding index reflecting RNA–RNA interaction and/or proximity detected between two distant RNA fragments, or an RNA is more extensively folded (Supplementary Fig. 10a,b). To calculate the folding index of a given transcript (or region), we computed the mean value of folding indexes for all chimeric reads mapped to this transcript (or region) as the transcript-level (or region-level) folding index. For transcriptome-wide comparisons, we plotted the distribution of folding index for all chimeric reads or the distribution of all transcript-level folding indexes. Sequencing depth differences and changes in the abundance of certain transcripts show minimal effects on the comparison of folding index between different conditions (Supplementary Fig. 10c–e). The median folding index for nuclear mRNA is 0.51, which is largely higher than that for the total cellular mRNA (median: 0.37; Supplementary Fig. 10f), confirming more extensive folding of nuclear RNA.

### Certain RBPs are associated with RNA–RNA interactions

RNA secondary structures have been demonstrated to drive RBP binding^24^, but the relationship between higher-order RNA structures and RBPs has yet to be fully explored. We first examined the association between RBP and RNA–RNA interactions by measuring RBP density at interaction regions using large-scale eCLIP data from ENCODE^25^. A larger number of RBP eCLIP peaks was observed on RNA–RNA interaction regions (identified using refolded RNA) than on shuffled regions in HepG2 and K562 cells (Supplementary Fig. 11a,b). We next quantified the association between individual RBP and RNA–RNA interactions by applying a multiple linear regression to correlate eCLIP reads density of each RBP to region-level folding index values throughout the K562 transcriptome. A small set of RBPs, including LIN28B, SRSF1, FXR1, FXR2, FMR1, SND1, METAP2, BUD13, ZNF622, UPF1 and YBX3, was identified to be positively correlated with RNA–RNA interactions (Supplementary Fig. 11c). The low correlation coefficients suggest a weak association, indicating that each RBP binds only to a small portion of RNA–RNA interaction regions.

To validate this observation, we performed KARR-seq in K562 cells after *YBX3* or *SRSF1* knockdown (Supplementary Fig. 11d,e). Knockdown of *YBX3* or *SRSF1* did not lead to obvious higher-order structure variations on their target RNAs (Supplementary Fig. 11f,g) nor transcriptome-wide changes of the folding index (Supplementary Fig. 11h). Therefore, each RBP seems to be associated with or regulate only a small number of RNA–RNA interactions, potentially due to their binding preferences to specific sequence and secondary structure motifs.

### Translation suppresses mRNA long-range interactions

**Fig. 3:**
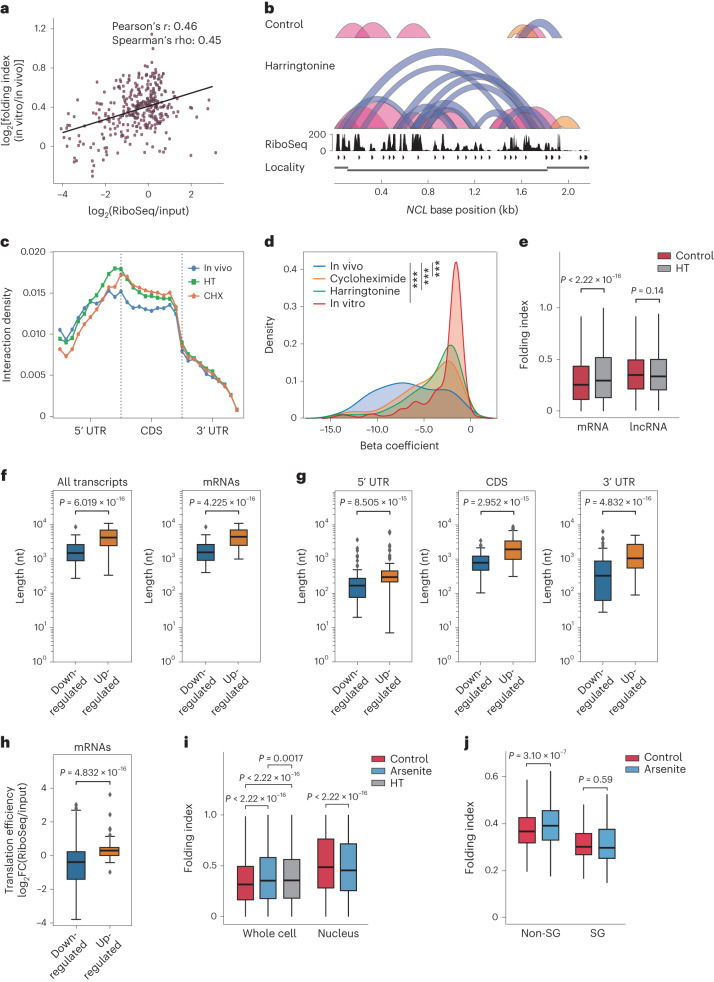
Translation suppresses mRNA higher-order structures under native and stress conditions. **a**, The effect of ribosome binding on RNA–RNA interactions in HepG2 cells. The *x* axis denotes ribosome binding strength, and the *y* axis shows the folding index difference between in vitro and in vivo. **b**, KARR-seq arc groups for the *NCL* transcript in control and harringtonine-treated HepG2 cells. Folding index: 0.246 for control and 0.290 for harringtonine. **c**, Metagene plot showing the relative abundance of intermolecular mRNA interactions under denoted conditions. CHX, cycloheximide; HT, harringtonine. **d**, The transcriptome-wide distribution of beta coefficients under denoted conditions. *** indicates *P* < 0.001. **e**, Folding index for mRNA and lncRNA in control and harringtonine-treated HepG2 cells. **f**, The length of transcripts that exhibit upregulated and downregulated intramolecular interactions after arsenite treatment in K562 cells. **g**, The 5′ UTR, CDS and 3′ UTR length for mRNAs that exhibit upregulated and downregulated intramolecular interactions after arsenite treatment in K562 cells. **h**, The translation efficiency under the normal condition for mRNAs that exhibit upregulated and downregulated intramolecular interactions after arsenite treatment in K562 cells. In **f**–**h**, for the analysis of all transcripts, *n* = 104 transcripts for the downregulated group and *n* = 73 transcripts for the upregulated group. For the analysis of mRNAs, *n* = 102 transcripts for the downregulated group and *n* = 68 transcripts for the upregulated group. **i**, mRNA folding index in control, arsenite-treated and harringtonine-treated K562 cells and purified K562 nuclei. *n* refers to the number of chimeric read level folding index. *n* = 440,484 for whole cell control, *n* = 242,268 for whole cell arsenite, *n* = 251,601 for whole cell HT, *n* = 154,797 for nuclear control and *n* = 162,507 for nuclear arsenite. **j**, mRNA folding index for SG-localized transcripts and other (non-SG) transcripts in control and arsenite-treated K562 cells. *n* = 161 transcripts in the non-SG group and *n* = 215 transcripts in the SG group. For **f**–**h**, *P* values were calculated by the one-sided Mann–Whitney test. For **e**,**i**,**j**, *P* values were calculated by the two-sided Mann–Whitney test. In box plots shown in **e**–**h**, the lower and the upper bounds denote 25th and 75th percentiles, respectively. The minima denote the lower bound −1.5× IQR. The maxima denote the upper bound +1.5× IQR. KARR-seq was performed in two biological replicates. IQR, interquartile range.

We next reasoned that ribosome translocation during translation could remodel higher-order RNA structures by resolving RNA duplexes. To test this hypothesis, we calculated the folding index difference between in vitro and in vivo conditions for each transcript. We observed a positive correlation between the folding index difference and ribosome occupancy density, suggesting that higher translation efficiency could lead to a larger difference in RNA folding between in vitro and cellular conditions (Fig. 3a). We then performed KARR-seq after treating HepG2 cells with translation inhibitors, harringtonine and cycloheximide. KARR-seq arc groups and interaction maps showed more intramolecular contacts upon translation inhibition (Fig. 3b and Supplementary Fig. 12a). Most harringtonine-induced and cycloheximide-induced interactions are located at CDS of mRNA (Fig. 3c), the region where ribosome translocation occurs during translation.

Consistently, inhibitor treatments right-shifted the distribution of RNA beta coefficients (Fig. 3d) and increased the averaged mRNA folding index (Fig. 3e). Meanwhile, translation inhibition did not alter the folding index for long non-coding RNAs (lncRNAs) (Fig. 3e), in line with the absence of translation machinery acting on these transcripts. These results collectively suggest that translation could resolve mRNA intramolecular interactions, leading to more stretched mRNA conformations. To validate this effect, we employed fluorescence in situ hybridization (FISH) imaging to measure the spatial distance between mRNA 5′ ends and 3′ ends under control and translation inhibition conditions. The 5′–3′ distances were around 100 nm for all three tested transcripts (Supplementary Fig. 12c), consistent with findings from previous single-molecule studies^26^. Translation inhibition resulted in shorter 5′–3′ distances in all three cases (Supplementary Fig. 12c), confirming that translation could indeed contribute to less extensive mRNA folding.

mRNA was proposed to form a ‘closed-loop’ structure mediated by translation initiation and termination complexes^27^. However, recent studies suggested a revised model of mRNA 5′–3′ communication^26,28,29^. Using KARR-seq data, we detected modest amounts of chimeric reads between mRNA 5′ and 3′ ends (Supplementary Fig. 12d). Intriguingly, 5′–3′ interactions were not more prevalent than *cis*-mRNA interactions at other regions (Supplementary Fig. 12e), and translation inhibition increased the proportion of 5′–3′ chimeric reads among all chimeric reads (Supplementary Fig. 12d). Consistent with recent reports^26,28^, our data suggest that mRNA loops are not completely closed, and translation inhibition may facilitate closer proximity between mRNA ends. Because mRNA loops are proposed to be mediated by large protein complexes, it is also possible that the sizes of dendrimers are not sufficient to capture all these interactions.

### Remodeling of higher-order RNA structures under arsenite stress

Extracellular stresses could remodel RNA homeostasis and induce stress granules (SGs)^30^; nonetheless, how stresses affect higher-order RNA structures and how RNA interactomes contribute to SG assembly remain to be investigated^31^. We conducted KARR-seq using K562 cells treated by sodium arsenite that induces oxidative stresses and SG formation. We performed differential RNA–RNA interaction analysis between normal and arsenite conditions (Supplementary Data 1). After arsenite treatment, transcripts bearing upregulated intramolecular interactions are much longer than those bearing fewer intramolecular interactions (Fig. 3f). mRNAs bearing upregulated intramolecular interactions after arsenite treatment possess longer 3′ UTR, CDS and 5′ UTR (Fig. 3g). These mRNAs also show a higher translation efficiency under the normal condition (Fig. 3h). Collectively, these results suggest the importance of RNA length and mRNA translation efficiency in remodeling higher-order RNA structurome under stress conditions.

Transcriptome-wide analysis revealed that arsenite treatment increased the mRNA folding index to a level seen in harringtonine-treated cells (Fig. 3i), indicative of more long-range RNA–RNA interactions and more extensive mRNA folding. Because arsenite is known to induce translation repression, this observation corroborates the suppressive effect of translation to higher-order RNA structures in a real physiological context. Interestingly, arsenite treatment decreased mRNA folding index in the nucleus (Fig. 3i), which might be attributed to the change of RBP composition in the nucleus or other secondary effects.

We next categorized mRNAs into two groups based on their localizations revealed by published SG transcriptomics data^24,32^. SG-localized transcripts show a lower averaged folding index than those that are not localized to SG (referred to as non-SG; Fig. 3j) under normal and arsenite conditions. Non-SG transcripts demonstrate an increased folding index after arsenite treatment, whereas the folding index of SG-localized transcripts remains unchanged (Fig. 3j). We envision that less extensive mRNA folding enhances the accessibility of mRNAs to interact with RBPs and other RNA molecules, which is crucial to the assembly of multi-component messenger RNP (mRNP) complexes within SGs. Indeed, the binding targets of SG marker proteins G3BP1 and TIA1 (refs. ^33–35^) showed slightly lower folding indexes under both conditions (Supplementary Fig. 12f). However, the small difference in folding indexes between G3BP1 targets and other transcripts suggests the existence of additional factors that determine RNA composition within SGs.

### KARR-seq identifies functional intermolecular RNA–RNA interactions

**Fig. 4:**
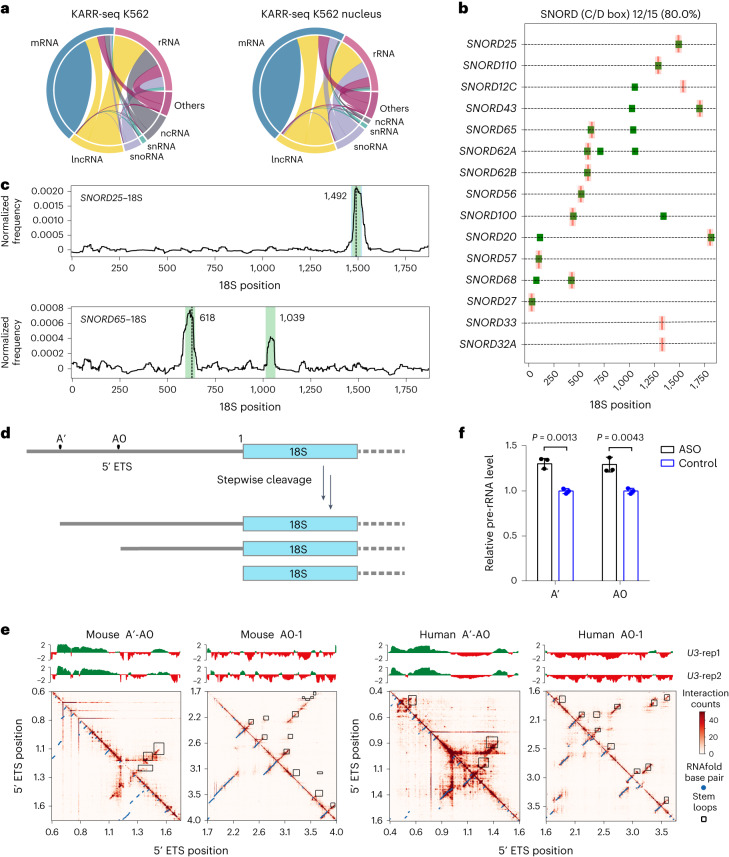
KARR-seq identifies functional RNA–RNA interactions between diverse RNA categories. **a**, The landscape of intermolecular RNA–RNA interactions revealed by KARR-seq in K562 cells (left) and K562 nucleus (right). The width of the link between two RNA categories denotes the relative abundance of chimeric reads taken by interactions between these two categories. mRNA–rRNA interactions, which are primarily a result of translation, were excluded from the plots. **b**, Interactions between C/D box snoRNA and 18S in K562 cells. Previously identified interaction sites are shown in pink. Interaction sites identified by KARR-seq are shown in green. **c**, Snapshots of KARR-seq data revealing *SNORD25*–18S and *SNORD65*–18S interactions. Regions colored in green denote identified interaction regions. The dashed lines denote previously known 2′ OMe modification sites. **d**, Scheme showing the organization and processing of human pre-rRNA 5′ ETS. **e**, Top, KARR-seq reads density for interactions between U3 and 5′ ETS in K562 cells (human) and mESCs (mouse). Bottom, KARR-seq interaction maps showing the higher-order structures of 5′ ETS in the corresponding cell lines. Stem loops are enclosed in black squares. **f**, Relative pre-rRNA levels in K562 cells treated by ASO that blocks a U3 interaction site at the 5′ ETS. Two sets of primers amplifying A′ and A0-proximal regions were applied for qPCR, respectively. Data are mean ± s.d. *P* values were calculated by Student’s *t*-test. *n* = 3 biological replicates. KARR-seq was performed in two biological replicates.

We next analyzed the intermolecular chimeric reads to identify RNA–RNA interactions between different RNA molecules. KARR-seq detects intermolecular interaction between various RNA categories in both intact cells and cell nuclei, such as lncRNA–mRNA, snoRNA–mRNA, snoRNA–rRNA and mRNA–mRNA interactions, with mRNA-mediated interactions taking the largest portion (Fig. 4a). KARR-seq and PARIS data reveal similar intermolecular interaction landscapes (Supplementary Fig. 13a,b), whereas snRNA, snoRNA and some other non-coding RNA species are depleted in RIC-seq data (Supplementary Fig. 13c).

To assess the sensitivity of KARR-seq in detecting functional intermolecular interactions, we enumerated interactions between C/D box snoRNA and rRNA in HepG2 cells and overlapped these interactions with previously identified rRNA 2′-*O*-methylation (2′-OMe) sites from the snoRNA database (snoRNA-LBME-db)^36^. snoRNA–rRNA interactions detected by KARR-seq overlap with 80% (12/15) of known modification sites on 18S (Fig. 4b,c) and 85% (34/40) on 28S (Supplementary Fig. 13d,e), suggesting a high sensitivity of KARR-seq. We also identified eight uncharacterized snoRNA–rRNA interactions on 18S (Fig. 4b,c) and 74 on 28S (Supplementary Fig. 13d,e). We experimentally validated these snoRNA–18S interactions by applying biotinylated antisense oligos (ASOs) that target regions adjacent to these identified interaction sites to pull down specific 18S rRNA fragments. A random biotinylated ASO was used as a control. The input and pull-down samples were subsequently subjected to reverse transcription–quantitative polymerase chain reaction (RT–qPCR) to amplify the corresponding snoRNAs. In four out of the six tested interactions, we observed enrichment of snoRNAs in the 18S pull-down samples compared to the control pull-down, suggesting the validity of these interactions (Supplementary Fig. 13f). These interactions may correspond to potential 2′-OMe sites that are not yet documented or suggest snoRNA functions beyond 2′-OMe deposition. The other two interactions, *SNORD65*–18S and *SNORD12C*–18S, could be false positives (Supplementary Fig. 13f).

### KARR-seq detects RNA–RNA interactions that affect pre-rRNA processing

rRNA maturation involves stepwise spacer cleavage from the polycistronic 45S pre-rRNA, which relies on extensive interactions between pre-rRNA and U3 RNA^37^. Several U3 binding sites on the 5′ external transcribed spacer (ETS) have been identified in bacteria and lower eukaryotes biochemically^38–41^, but the landscape of U3–pre-rRNA interactions within mammalian cells and how these interactions influence the 5′ ETS structure are unclear.

The 5′ ETS of mammalian pre-rRNA harbors three closely located cleavage sites, namely A′, A0 and 1 (Fig. 4d)^37^. KARR-seq revealed extensive interactions between U3 and the A′-A0 region in both K562 cells and mESCs, whereas minimal interactions were detected at the A0-1 region (Fig. 4e, top). In the meantime, A′-A0 and A0-1 regions show distinct higher-order structure features: A′-A0 forms highly dynamic stripe and domain structures, whereas A0-1 includes an array of stable stems (Fig. 4e, bottom). The strength of U3–rRNA interaction tends to decrease as intramolecular 5′ ETS interactions become pronounced (Fig. 4e). Therefore, we propose that U3 RNA may regulate 5′ ETS processing by maintaining the correct conformation of A′-A0 through direct U3–pre-rRNA interactions. The A0-1 region is less involved in U3-mediated interactions because its stem structures are relatively stable and less susceptible to conformational changes. ASO (Methods) that blocks U3–ETS interactions at the A′-A0 region increased pre-rRNA level in HepG2 cells compared to the control ASO (Fig. 4f), suggesting the importance of U3–ETS interaction in regulating rRNA biogenesis in mammalian cells.

### KARR-seq detects RNA–RNA interactions in virus-infected cells

Many viruses use RNA to store genetic information. These viruses have evolved extensively to regulate their life cycle through RNA structure-based mechanisms and can efficiently harness host cellular machineries^42,43^. The higher-order structures of most viral genomes and RNA–RNA interactions between virus and host are largely unexplored. In light of this, we applied KARR-seq to A549 cells infected by human RSVs and VSVs, respectively. RSV is a prominent cause of respiratory tract infection in infants, children, the elderly and immunocompromised individuals^44^, whereas VSV has been used for decades as a model system for negative-sense RNA viruses^45^.

**Fig. 5:**
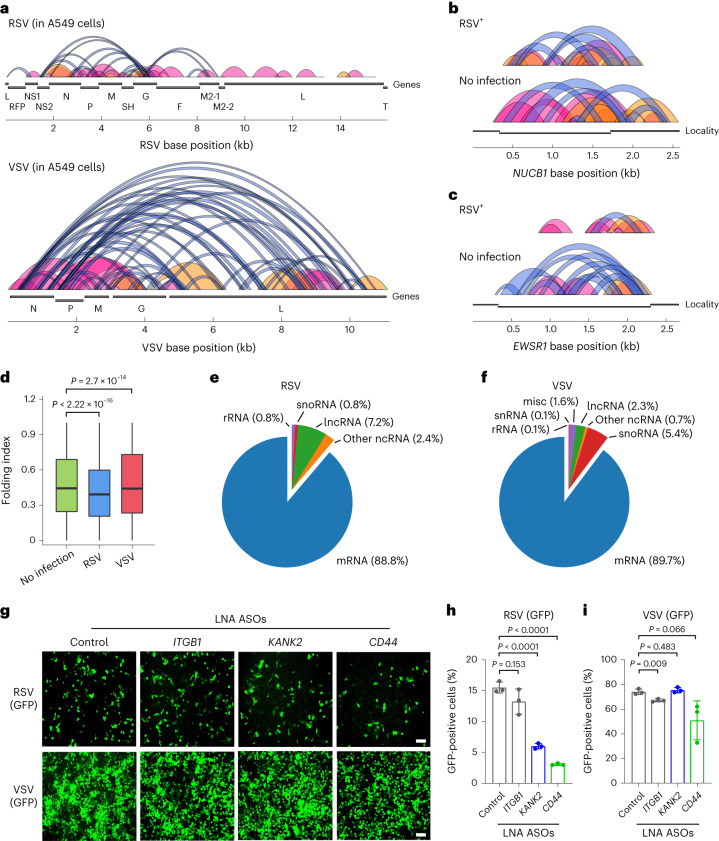
KARR-seq reveals viral RNA structures and virus–host RNA–RNA interactions. **a**, Loop and stripe structures across the RSV (top) and VSV (bottom) RNAs in infected A549 cells. **b**,**c**, KARR-seq arc groups for the *NUCB1* (**b**) and *EWSR1* (**c**) transcripts in control and RSV-infected A549 cells. Folding index: 0.415 for *NUCB1* after RSV infection, 0.527 for *NUCB1* without infection, 0.411 for *EWSR1* after RSV infection and 0.532 for *EWSR1* without infection. **d**, RNA folding index in control, RSV-infected and VSV-infected A549 cells. *n* denotes the number of chimeric read level folding index. *n* = 1,772,734 for no infection, *n* = 596,451 for RSV and *n* = 159,725 for VSV. The lower and the upper bounds denote 25th and 75th percentiles, respectively. The minima denote the lower bound −1.5× IQR. The maxima denote the upper bound +1.5× IQR. *P* values were calculated by the two-sided Mann–Whitney test. **e**,**f**, The number of host RNAs from each RNA category that interact with RSV (**e**) and VSV (**f**) RNAs. **g**, Fluorescent imaging of GFP-tagged RSV and GFP-tagged VSV after cells were transfected with denoted LNA ASOs. These ASOs target mRNA transcripts at positions that interact with RSV RNA. Scale bar, 100 µm. **h**,**i**, The percentage of RSV-positive (**h**) and VSV-positive (**i**) cells quantified by flow cytometry after cells were treated with denoted LNA ASOs. Data are mean ± s.d. *n* = 3 biologically replicates. *P* values were calculated by two-tailed Student’s *t*-test. IQR, interquartile range.

KARR-seq coverage on VSV is roughly five times the coverage on RSV. KARR-seq data revealed three layers of information: (1) higher-order structures of RSV and VSV RNAs; (2) effects of virus infection on the higher-order structures of the host transcriptome; and (3) RNA–RNA interactions between viral and host RNAs. Both RSV and VSV are non-segmented negative-sense RNA viruses and share a similar genome organization. However, one unique feature in the RSV RNA genome is the presence of a G/C-rich *G* gene. KARR-seq detected higher-order structures of both RSV and VSV RNAs. Interestingly, within the RSV RNA, identified interactions are clustered around the *G* gene and are mostly short-ranged (<2 Kb) (Fig. 5a, upper panel). In contrast, the VSV RNA includes substantially more long-range stem–loop interactions (Fig. 5a, lower panel).

We next analyzed how RSV and VSV infection could affect the higher-order structures of the host transcriptome. We found that RSV infection resulted in a reduction of intramolecular interactions of the host mRNA in A549 cells (Fig. 5b–d). This effect was not observed in VSV-infected cells. Because translation could suppress global mRNA compaction, the differences between mRNA intramolecular interactions in RSV-infected and VSV-infected cells are potentially related to a rapid host translation shutdown upon VSV but not RSV infection^46,47^.

Both RSV and VSV RNAs interact with host mRNAs and ncRNAs (Fig. 5e,f). Host microRNAs and small nuclear RNAs (snRNAs) have been demonstrated to bind viral genomes to regulate virus life cycles and host RNA metabolism^7,48^. However, interactions between viral RNA and host mRNAs have not been well documented. KARR-seq detected 111 and 664 host mRNA transcripts that interact with the RSV and VSV RNAs, respectively (Supplementary Fig. 14a,b). Different from the severe acute respiratory syndrome coronavirus 2 (SARS-CoV-2) RNA that preferentially interacts with the CDS of host mRNAs^49^, both RSV-mediated and VSV-mediated interactions are enriched at the 3′ UTR of host mRNAs (Supplementary Fig. 14c). The interactions between RSV and VSV RNAs with host RNAs predominantly occurs through *N*, *G* and *L* genes (Supplementary Fig. 14d,e). Although RSV and VSV infections activate similar pathways in A549 cells (Supplementary Fig. 14f,g), mRNA transcripts that interact with RSV and VSV RNAs enriched distinct functions. RSV-interacting transcripts are involved in responses to cytokine and regulation of apoptosis (Supplementary Fig. 14h), whereas VSV-interacting transcripts regulate RNA processing, translation, decay and protein targeting (Supplementary Fig. 14i).

We next investigated the potential functional relevance of host mRNA–RSV interactions. We focused on mRNAs that are related to cytokine-mediated signaling and apoptosis and designed ASOs that contain locked nucleotides (LNA ASOs) to target these mRNAs in A549 cells, to block specific RNA–RNA interactions. We infected the cells with GFP-tagged RSV or VSV and assayed virus replication by measuring GFP signals in ASO-treated cells. As shown by fluorescent imaging and quantification by flow cytometry, LNA ASOs targeting *KANK2* and *CD44* mRNA repressed RSV replication by more than 60% (Fig. 5g,h). In the meantime, these ASOs showed minor effects on VSV replication (Fig. 5g,i), supporting that the repression effect is RSV specific and likely from the blockage of the corresponding RNA–RNA interactions. These results confirmed the capability of KARR-seq in identifying functional intermolecular RNA–RNA interactions in infectious disease models. Instead of passively binding to the most abundant host transcripts, different viral RNAs interact with host mRNA with diverse functions, suggesting roles of virus-specific RNA–RNA interactions in regulating virus propagation. Future systematic studies are required to reveal the exact molecular mechanisms.

## Discussion

How different RNA molecules interact with each other and assemble into higher-order structures has been a long-standing question. Psoralen-mediated and RBP-mediated approaches have been widely applied with successes^3–11^; nonetheless, complementary methods to improve sensitivity and analyze RNAs in all cellular compartments are still in demand. A SHAPE-based bi-functional crosslinker was recently developed to map RNA–RNA interactions, but its optimal application was observed in a locus-specific manner using purified RNA in vitro^50^. Taking advantage of kethoxal-mediated RNA functionalization and multifunctional chemical crosslinkers, we developed KARR-seq to detect higher-order RNA structures and pairwise interactions between various RNA categories. KARR-seq not only captures stable RNA base-pairing contacts but also identifies transient RNA–RNA interactions or proximity that do not correspond to secondary structures. Although we focused on RNA proximity in this work, the dendrimer-mediated crosslinking platform could have other applications, such as studying RNA–chromatin interactions and the mechanisms of RNA-mediated compartment assembly^51^.

We noticed that the median physical distances captured by G1 and G7 do not always match the diameters of the dendrimers. In addition, the KARR-seq contact frequency map and the cryo-EM physical distance map of 18S show evident differences. Although the cryo-EM structures reveal the most thermodynamically stable conformations in vitro, RNAs may fold differently in live cells. Given the transient and dynamic nature of many RNA–RNA interactions within cells, it is plausible that these interactions might be effectively captured only when the sizes of crosslinkers match the spatial distances between two RNA fragments. For large dendrimers such as G7, we attached DBCO to multiple surface amine groups. The distance between two DBCO groups can be smaller than the diameter of the dendrimer, enabling the crosslinking of RNAs at closer proximities.

KARR-seq identifies intermolecular RNA–RNA interactions between various RNA categories, including those responsible for the maintenance of pre-rRNA processing and those that may regulate virus metabolism in the host cells. These examples demonstrate the value of KARR-seq in revealing RNA functions through RNA–RNA interactions. The molecular bases behind these functions require comprehensive mechanistic studies. When coupled with protein or RNA enrichment techniques, KARR-seq can be further tailored to study specific biological processes by enriching rare RNA–RNA interactions mediated by specific RBPs and transcripts.

## Methods

### Cell culture

A549 (American Type Culture Collection (ATCC), CCL-185), HEp-2 (ATCC, CCL-23), Vero CCL81 (ATCC, CRLCCL81), HEK293T (ATCC, CRL11268) and HepG2 (ATCC, HB8065) cells were maintained in DMEM (Gibco, 11995) with 10% (v/v) FBS (Gibco) and penicillin–streptomycin. F123 mESCs (Bing Ren laboratory, University of California, San Diego) were cultured in DMEM (Gibco, 11995) with 10% (v/v) FBS, 1 mM L-glutamine (Gibco), 0.1 mM β-mercaptoethanol (Gibco), 1% (v/v) non-essential amino acid stock (100×, Gibco), 1,000 U ml^−1^ LIF (Millipore) and penicillin–streptomycin. K562 cells (ATCC, CCL243) were cultured in RPMI 1640 (Gibco 11875) with 10% (v/v) FBS and penicillin–streptomycin. *Drosophila* S2 cells (Thermo Fisher Scientific, R69007) were maintained in Schneider’s *Drosophila* media supplemented with 10% (v/v) FBS, 0.1% Pluronic F-68 (Gibco) and penicillin–streptomycin. *Drosophila* S2 cells were grown at 28 °C. The other cells were grown at 37 °C with 5% CO_2_.

To inhibit translation, cells were treated with 2 µg ml^−1^ harringtonine (Abcam, ab141941) or 100 µg ml^−1^ cycloheximide (Abcam, ab120093) for 30 min before being harvested. To induce SGs, K562 cells were treated with 200 µM sodium arsenite (Sigma-Aldrich, S7400) for 1 h before being harvested. siRNAs were transfected using Lipofectamine RNAiMAX (Invitrogen, 13778150). After transfection, cells were cultured for 2–3 d before being harvested.

### Virus inoculation

Recombinant human RSV A2 strain expressing GFP (rgRSV) was grown and titered in HEp-2 cells. VSV Indiana strain expressing GFP (rVSV-GFP) was grown and titered in Vero CCL81 cells. Confluent A549 cells in T25 flasks were infected with rgRSV or rVSV-GFP at a multiplicity of infection (MOI) of 0.1. After 1 h of adsorption, the inoculum was removed, and fresh DMEM with 2% FBS was added. Infected cells were inoculated at 37 °C. rVSV-GFP-infected cells were fixed at 24 h after infection, and rgRSV-infected cells were fixed at 48 h after infection for KARR-seq.

### Western blot

Protein samples were subjected to SDS-PAGE and transferred to a nitrocellulose membrane (Bio-Rad). The membranes were then blocked in 5% milk in PBS with 0.1% Tween 20 (PBST) and subjected to overnight incubation with primary antibodies in 5% milk in PBST at 4 °C. Membranes were washed five times with PBST and then incubated with secondary antibodies for 1 h at room temperature. Membranes were then washed five times before being applied with enhanced chemiluminescence (ECL) and developed. Antibody information is as follows: rabbit anti-SRSF1 (Bethyl Laboratories, A302-052A, 1:1,000); rabbit anti-histone H3 (Cell Signaling Technology, 9717, 1:1,000); rabbit anti-β-tubulin (Cell Signaling Technology, 2146, 1:1,000); rabbit anti-U1-70K (Abcam, ab83306, 1:1,000); rabbit anti-YBX3 (GeneTex, GTX130052, 1:1,000); mouse anti-GAPDH (Proteintech, HRP-60004, 1:20,000); and anti-rabbit IgG, HRP-linked (Cell Signaling Technology, 7074, 1:2,500).

### Dot blot

Purified RNA (1 µl) was loaded onto the Amersham Hybond-N+ membrane (GE Healthcare, RPN119B). Membranes were air dried and crosslinked twice using UV Stratalinker 2400 at 150 mJ cm^−^^2^. Membranes were then blocked overnight in 5% fatty-acid-free BSA in PBST. Membranes were then washed and incubated in streptavidin-HRP (Thermo Fisher Scientific, S-911) in PBST supplemented with 3% fatty-acid-free BSA. The membrane was washed five times before being applied with ECL and developed.

### Synthesizing DBCO and biotin-modified dendrimers

PAMAM dendrimer G1 (1.53 µmol; Sigma-Aldrich, 412384), DBCO-NHS (3.06 µmol; Sigma-Aldrich, 761524), biotin-NHS (1.53 µmol; Sigma-Aldrich, 203112) and triethylamine (5 µl; Sigma-Aldrich, 471283) were added to 2 ml of methanol. The reaction mixture was stirred overnight at room temperature before 100 µl of acetic anhydride (Sigma-Aldrich, 320102) and 100 µl of triethylamine were added. The reaction mixture was stirred for another 24 h at room temperature. The dendrimer solution was purified and concentrated with Microsep Advance Centrifugal Devices with Omega Membrane 1K (Pall Corporation, MCP001C41). DBCO-modified and biotin-modified G3, G5 and G7 were synthesized following a similar procedure, with four equivalent DBCO added for G3, 16 equivalent DBCO added for G5 and 64 equivalent DBCO added for G7.

Characterization and quantification of modified dendrimer were performed by measuring the characteristic UV absorbance of the DBCO moiety at 295 nm. A series of DBCO-NHS solutions with known concentrations were prepared as external standards to generate a calibration curve.

### KARR-seq

A detailed KARR-seq protocol is included as Supplementary Protocol. In brief, cells were crosslinked in 1% formaldehyde for 10 min. For each KARR-seq sample, 2–5 million cells were resuspended into 500 µl of permeabilization buffer (10 mM Tris-HCl, pH 8.0, 10 mM NaCl, 0.2% IGEPAL CA-630, 5 mM EDTA) supplemented with 2 mM N_3_-kethoxal, proteinase inhibitor and RNase inhibitor. Rotate the mixture at room temperature for 30 min. The cells were washed once with permeabilization buffer and then resuspended in 500 µl of permeabilization buffer supplemented with 12.5 µM dendrimers. The click reaction was performed at 37 °C for 1 h with shaking. To isolate RNA, wash and resuspended cell pellets in 410 µl of 25 mM K_3_BO_3_, 50 µl of 10% SDS, 30 µl of proteinase K (Thermo Fisher Scientific, 25530049) and 10 µl of SUPERNase inhibitor (Thermo Fisher Scientific, AM2696). The mixture was shaken at 55 °C for 2 h and subjected to phenol–chloroform extraction and ethanol precipitation.

Dissolve precipitated RNA in 105 µl of 25 mM K_3_BO_3_, 12 µl of 10× DNase I buffer (Thermo Fisher Scientific, AM8170G), 2 µl of DNase I (Thermo Fisher Scientific, 18047019) and 1 µl of SUPERNase inhibitor. The mixture was gently shaken at 37 °C for 30 min. Then, 130 µl of 2× proteinase K buffer (100 mM Tris-HCl, pH 7.5, 200 mM NaCl, 2 mM EDTA, 1% SDS) and 10 µl of proteinase K were then added. The mixture was shaken at 55 °C for another 30 min, followed by phenol–chloroform extraction and ethanol precipitation. Precipitated RNA was dissolved in a mixture of 63 µl of 25 mM K_3_BO_3_ and 7 µl of RNA fragmentation buffer (Thermo Fisher Scientific, AM8740). The mixture was heated at 70 °C for 15 min before the reaction was quenched.

For each sample, block 30 µl of Dynabeads Myone Streptavidin C1 (Thermo Fisher Scientific, 65001) at room temperature with 100 µl of 1× binding/wash buffer (5 mM Tris-HCl, pH 7.4, 0.5 mM EDTA, 1 M NaCl, 0.05% Tween 20) containing 1 µg µl^−1^ BSA (New England Biolabs (NEB), B9000S) and 1 µg µl^−1^ salmon sperm DNA (Thermo Fisher Scientific, 15632011) for 30 min. Beads were washed and resuspended in 80 µl of 2× binding/wash buffer (10 mM Tris-HCl, pH 7.4, 1 mM EDTA, 2 M NaCl, 0.1% Tween 20) and incubated with the fragmented RNA at room temperature for 20 min with rotation. Beads were washed twice with 100 µl of 1× binding/wash buffer and once with 100 µl of 1× PNK buffer (diluted from 10×; NEB, M0201L).

Resuspend the beads in 41 µl of 25 mM K_3_BO_3_. Add 5 µl of 10× T4 PNK buffer, 3 µl of T4 PNK (NEB, M0201L) and 1 µl of SUPERNase inhibitor. Shake the beads at 37 °C for 30 min. Then, add 1 µl of 10× T4 PNK buffer, 3 µl of T4 PNK and 6 µl of 10 mM ATP. Shake the tube at 37 °C for another 30 min. The beads were then washed twice with 1× binding/wash buffer and once with 1× ligation buffer (diluted from 10×; NEB, M0437M). Resuspend beads in 673 µl of 25 mM K_3_BO_3_, add 100 µl of 10× T4 RNA ligase buffer, 2 µl of 10 mM ATP, 200 µl of 50% PEG 8000, 20 µl of T4 RNA ligase I (NEB, M0437M) and 5 µl of SUPERNase inhibitor. Shake the reaction mixture at 16 °C overnight.

After ligation, wash beads three times with 1× binding/wash buffer. Elute RNA by heating the beads in 50 µl of water at 95 °C for 10 min. Purify the RNA using the RNA Clean & Concentrator Kit (Zymo Research, R1014). Then, 10 ng RNA was used for library construction using the SMARTer Stranded Total RNA-Seq Kit v2–Pico Input Mammalian (Takara, 634413). Libraries were sequenced on the Illumina NovaSeq platform, PE150 mode, with around 100 million reads per sample.

### Cell nuclei isolation

Ten million K562 cells were collected and washed once with 1 ml of ice-cold PBS with 1 mM EDTA. Then, 200 μl of ice-cold lysis buffer (10 mM Tris-HCl, pH 7.5, 0.1% NP40, 150 mM NaCl) was added to washed cell pellet, followed by incubation on ice for 5 min. Next, 500 μl of chilled sucrose cushion (24% sucrose in lysis buffer) was gently added below the lysate. The tube was centrifuged at 4 °C at 15,000*g* for 10 min, and the supernatant was discarded. Then, 200 μl of ice-cold PBS with 1 mM EDTA was gently added to the nuclei pellet without dislodging the pellet and was then aspirated. Around 5 million nuclei were used for each KARR-seq experiment. Nuclei were crosslinked as described above.

### KARR-seq data processing

SeqPrep was first used to merge overlapping read pairs into single-end reads. Reads that failed to merge were excluded from the analysis. The resulting FASTQ files were mapped to both the genome and transcriptome (mm10, hg19 or dm3) using STAR with PCR duplications removed^52^. STAR were used to recover gapped and chimeric reads (--runMode alignReads --outFilterMultimapNmax 100 --outSAMattributes all --alignIntronMin 1 --scoreGapNoncan -4 --scoreGapATAC -4 --chimSegmentMin 15 --chimJunctionOverhangMin 15 --limitOutSJcollapsed 10000000 --limitIObufferSize 1500000000). Gapped reads were defined as reads containing at least one N entry in the CIGAR string. For alignments done using the reference genome, an extra filtering step was performed to remove gapped reads that overlap known splicing junctions. Gapped reads were combined with chimeric reads from the <sample>_Chimeric.out.sam output file to form the final chimeric reads set. Processed results were stored and indexed in the pairix format for efficient range-based querying. Identical processing steps described above were applied for RIC-seq data. For PARIS data, readCollapse.pl was performed to collapse reads with unique molecular identifier (UMI) barcodes. KARR-seq data processing statistics are included in Supplementary Table 1.

To identify differential chimeric groups between conditions, we used DESeq2 (ref. ^53^) and applied median normalization and Wald’s test. Differential chimeric groups were reported as significant if the adjusted *P* < 0.05.

### Comparison to physical distance maps

We used the 18S physical distance map to calibrate and infer the physical distance proximity. We calculated physical distances of 18S at a granularity of 5-nucleotide (nt) resolution by calculating the Euclidean distance (L2 normalization) between the centroid positions of each 5-nt window using the cryo-EM structure^20^. To tabulate the inferred physical distance distribution from KARR-seq, PARIS and RIC-seq, we looked up the physical distance value from the 5-nt pairwise physical distance table based on the midpoints from either arm of the 18S chimeric reads. A Gaussian kernel was applied for the kernel density estimate of the physical distance distribution.

For 28S, U3 and *TERC*, the consistency between physical distance maps and contact maps for each transcript was evaluated using ROC–AUC curves, with physical distance maps binarized using a gamma distribution. The gamma distribution was parameterized as α = 6 and β = µ/α, where the mean physical distance µ = 20 Å.

### Chimeric reads clustering and the identification of loops and stripes

To identify loops and stripes, we clustered chimeric reads and categorized them in an unsupervised fashion. We first calculated the pairwise distance using a custom overlapping similarity function:

$${\rm{Minimum}}\,{\rm{of}}\,{\rm{overlap}}({{\rm{left}}}_{1},{{\rm{left}}}_{2}){\rm{and}}\,{\rm{overlap}}({{\rm{right}}}_{1},{{\rm{right}}}_{2})$$

We then built a graph from the similarity matrix and detected clusters of chimeric reads to define RNA–RNA interactions using the Clauset–Newman–Moore modularity-based method. For each interaction, a minimum of three chimeric reads must be present to support clustering. Representative positions (left, right) of interactions were defined as interaction anchors. Left anchors were defined using the median of all left-arm starting positions, and right anchors were defined using the median of all right-arm ending positions.

Chimeric groups were clustered as stable structures (loops) and dynamic structures (stripes). Loops were defined as interactions with both interacting fragments having fixed positions or limited variability in their positioning. Stripes were defined as interactions with a fixed position on one end and variable interacting positions on the other end. To characterize loops and stripes, we computed the coefficient of variation (CV) for each anchor. Chimeric groups with CV < 1.0 on both anchors were defined as loops. Chimeric groups with CV < 1.0 only on the right or left anchor were defined as right or left stripes, respectively.

### Secondary structure prediction by RNAcofold

RNAcofold (ViennaRNA version 2.4.3) was used to make secondary structure predictions and calculate their associated MFE values at interaction anchors. For comparison with the MFE calculated at all interaction anchors, a background set was generated using BEDtools (version 2.26.0) to shuffle the anchor positions while maintaining the same span distribution and proportion of anchors present on the transcript (bedtools shuffle -chrom).

### Calculating folding index

The folding distance was defined as the genomic distance between the left arm (start position) of the chimeric read to the right arm (start position) of the chimeric read. To quantify the levels of RNA compaction and to account for differences between transcripts of varying lengths, abundance and experimental conditions, we devised a folding index, a bounded transformation of the span distance. We score the span distance between a bounded interval of 0 to 1, where a value of 0 suggests rigidity and 1 suggests a tendency to compact through folding.

$${\rm{Folding}}\,{\rm{index}}=1.0-{{\rm{e}}}^{-{\rm{\alpha }}{\rm\it{s}}}$$

where *s* is the span distance, and $\alpha =\frac{1}{200}$.

This is a monotonically increasing curve that increases linearly and as a function of span distance before converging to 1. It effectively down-weighs excessively long transcriptomic spans. α was parameterized to 1/200 to represent a 150-nt distance at folding index = 0.5. This metric can be applied to a certain transcript, a given region within a transcript or transcriptome wide.

### Ribosome profiling data processing

Pre-processing of ribosome profiling sequences was done using Cutadapt (cutadapt –q 30 –a AGATCGGAAGAGCACACGTCT –u 3 –minimum-length 10). The first three bases from the 5′ end were clipped. Sequences were then aligned using BWA with default parameters. Genome coverage files were created using BEDtools (bedtools genomecov –bga) and converted into UCSC bigWig format. Ribosome stalling sites were identified using a model-free approach with CTK^54^ (perl tag2peak.pl -big –ss –v –valley-seeking –valley-depth 0.9).

### Tertiary structure modeling

We modeled tertiary structures and calculated the expected distance based on the FJC model.

$$f(x)=4\uppi {x}^{2}{\left(\frac{1}{2\uppi {\sigma }^{2}}\right)}^{3/2}{\rm {e}}^{-\left(\frac{{x}^{2}}{2{\sigma }^{2}}\right)}$$

$${\sigma }^{2}=\frac{N{l}^{2}}{3}$$

where *x* is the end-to-end distance between two points.

Average end-to-end distance (E2E) between two points:

$${\rm {E2E}}=\sqrt{N}l$$

where *N* is the number of monomers/nucleotides between two points, and $l$ is the fixed length of an RNA nucleotide.

We incorporated secondary structure and MFE into our tertiary structure modeling. Using secondary structure dot-bracket notation, we first applied forgi’s BulgeGraph (ViennaRNA) class to construct the graph. We then constructed the two-dimensional (2D) tree graph, G_mst_, by finding the minimum spanning tree using Kruskal’s algorithm.

Next, we constructed expected physical distance maps of the tree graph by incorporating the E2E average physical distance function. The physical distance maps were used to restrain the polymer model constructed using the IMP framework^55^. The final polymer chain was output in (*x*,*y*,*z*) coordinates, and the simulated physical distance maps were derived using Euclidean distance.

### RBP eCLIP analysis

Processed eCLIP BAM files were retrieved from ENCODE. To determine the crosslinking-induced termination sites (CITSs), we summed the counts of the pileups flanking 7 nucleotides upstream and downstream of a given locus X_i_. For each transformed value T_i_, we estimated C_i_ as the local Poisson parameter and infer it using the means of C_i−100_ to C_i+100_. *P* values were then computed for each locus i and fitted to a beta-uniform mixture model to correct for multiple testing. For each locus i, a posterior probability was calculated to determine whether the *P* value is more likely to follow the beta (significant) or uniform (random) distribution. We used a posterior probability greater than 90% as the threshold for calling significant loci. Consecutive loci greater than the length of 15 were concatenated.

We called CITS on the respective eCLIP controls samples to remove artifacts. We merged the CITS calls by taking only their common intersecting region to yield a stringent eCLIP call set.

### Identification of snoRNA–rRNA interactions

We used BWA-mem (bwa mem -M) to map all chimeric segments to identify intermolecular interactions. Chimeric reads were aligned to the canonical transcriptome, with the longest spliced isoforms selected to represent corresponding gene entries. For each C/D box snoRNA, we computed the pile-up coverage of chimeric segments across the transcript length of either 18S or 28S using BEDtools (bedtools genomecov -bg). Regions with systematically high coverages across all snoRNA–rRNA pairs and did not overlap known 2′-OMe sites were deemed as backgrounds. SciPy’s find_peak function was applied to remove background and identify snoRNA–rRNA peaks using the following parameters: width = 50, distance = 50, height = 1 × 10^−4^.

### Differential gene expression and gene set enrichment analysis

For RSV-infected and VSV-infected samples, gene counts for mock and infected samples were tabulated using the non-chimeric reads of KARR-seq. Differential gene expression analysis was performed between mock and viral-infected samples using DESeq2 (ref. ^53^). Gene set enrichment analyses (GSEAs) were performed using GSEApy^56^ using transcripts with (1) host–viral interactions (with at least 100 host–viral chimeric reads) and (2) differentially expressed transcripts (adjusted *P* < 0.01 and log_2_ fold change (FC) > 1.5 and log_2_FC < −1.5).

### Preparation of FISH probes

FISH probes were designed using Stellaris Probe Designer (version 4.2) and purchased from Integrated DNA Technologies with 5′ amine modifications. For each transcript, we designed 20 probes to target each ends. Pooled probes targeting the 5′ end and the 3′ end were labeled with Alexa Fluor 647 (AF647) NHS Ester (Thermo Fisher Scientific, A20006) and Cy3B NHS ester (Cytiva, PA63101) following the previously published protocol^57^. Labeled probes were purified by ethanol precipitation, followed by running through P6 Micro Bio-Spin columns (Bio-Rad, 7326228). The probe concentration and labeling efficiency were determined using a BioSpectrometer (Eppendorf).

### RNA FISH

RNA FISH was performed following a published protocol^58^. HepG2 cells were first fixed and permeabilized. Then, 100 µl of 1 nM FISH probes diluted in the hybridization buffer (10% dextran sulphate and 10% formamide in 2× SSC) were applied to cells, and the cells were incubated for 16 h at 37 °C. The cells were then washed twice with FISH wash buffer (10% formamide in 2× SSC) for 30 min before imaging.

Samples were imaged in an imaging buffer containing 50 mM Tris-HCl (pH 8.0), 10% glucose, 2× SSC, 0.5 mg ml^−1^ glucose oxidase (Sigma-Aldrich) and 67 µg ml^−1^ catalase (Sigma-Aldrich). Imaging was performed using a Nikon TiE microscope with a CFI HP TIRF objective (×100, NA = 1.49, Nikon) and an EMCCD (Andor, iXon Ultra 888). Signals were acquired using a 647-nm laser (Cobolt MLD) and a 561-nm laser (Coherent Obis).

### RNA FISH data analysis

Spot detection was separately performed in the AF647 and Cy3B channels using the ImageJ (1.53n) plugin ThunderSTORM (version 1.3)^59^ in 2D. The ThunderSTORM was configured with the following parameters. Image filtering: wavelet filter with a third-order B-spline function and a scaling factor of 2; approximate localization of molecules: local maximum method with 8-neighborhood connectivity and a peak intensity threshold set to three times of the standard deviation of the first wavelet level; sub-pixel localization of molecules: PSF (Integrated Gaussian) method with a maximum likelihood fitting method, a fitting radius of 5 pixels (pixel size = 130 nm) and an initial sigma of 1.6 pixels; and XY uncertainty threshold: 40 nm.

Inter-RNA distances were subsequently calculated using a custom MATLAB (R2021b) code. Chromatic aberration was corrected using TetraSpeck Microspheres (Thermo Fisher Scientific). AF647 and Cy3B spot pairs with a center-to-center distance of less than 300 nm were considered signals from the same mRNA molecule.

### Analysis of pre-rRNA level after ASO blockage

Sequences for ASOs and qPCR primers are included in Supplementary Table 2. ASOs were delivered into K562 cells by electroporation using an SF Cell Line 4D-Nucleofector X Kit (Lonza, V4XC-2024). After 48 h, cells were collected for RNA purification using TRIzol reagent (Thermo Fisher Scientific, 15596026). Purified RNA was then applied to reverse transcription, followed by qPCR.

### Validation of snoRNA–18S interactions

Sequences for biotinylated ASOs and qPCR primers are included in Supplementary Table 2. K562 cells were treated as indicated in the KARR-seq procedure. Total RNA was then isolated from the cells, fragmented and purified as above. Five percent of the fragmented RNA was saved as input. The rest was split into portions and mixed with biotinylated ASOs targeting different rRNA regions in the hybridization buffer (75 mM HEPES, pH 7.0, 150 mM KCl, 25 mM K_3_BO_3_). The mixture was denatured, followed by a gradual temperature decrease to 25 °C for re-hybridization. The mixture was then subjected to enrichment using 30 µl of Dynabeads Myone Streptavidin C1 (Thermo Fisher Scintific, 65001). Enriched RNA and the corresponding input samples were subjected to RT–qPCR.

### Analysis of RSV and VSV replication after ASO blockage

ASOs (Supplementary Table 2) were transfected using Lipofectamine RNAiMAX. In brief, A549 cells in 24-well plates were transfected with 80 nM ASO for 24 h, followed by virus infection with rgRSV or rVSV-GFP at the MOI of 0.1. After incubation at 37 °C for 1 h, the virus inoculum was removed, and fresh DMEM with 2% FBS was added. The infected cells were incubated at 37 °C. GFP expression was monitored by fluorescence microscopy and flow cytometry using an Attune NxT Flow Cytometer (Thermo Fisher Scientific). Flow cytometry data were analyzed using FlowJo (10.0.7) software.

### Reporting summary

Further information on research design is available in the Nature Portfolio Reporting Summary linked to this article.

## Online content

Any methods, additional references, Nature Portfolio reporting summaries, source data, extended data, supplementary information, acknowledgements, peer review information; details of author contributions and competing interests; and statements of data and code availability are available at 10.1038/s41587-023-02109-8.

## Supplementary information

Supplementary Information Supplementary Figs. 1–16 and Protocol.

Reporting Summary

Supplementary Tables 1 and 2 Supplementary Table 1: Sequencing and mapping statistics. Supplementary Table 2: Oligonucleotides used in this work.

Supplementary Data Differential RNA–RNA interactions between arsenite and control conditions.

## Data Availability

Public sequencing data used in this study were acquired from the Gene Expression Omnibus (GEO) with accession numbers as follows: GSE127188 (RIC-seq, HeLa)^12^; GSE74353 (PARIS, HEK293T)^4^; GSE138058 (G3BP1-APEX-seq)^32^; and GSE121952 (ribosome profiling)^60^. Cryo-EM structures were acquired from the Protein Data Bank (accession codes: 7QXB for *TERC*^61^, 6QX9 for U3 (ref. ^62^) and 4V6X for 18S and 28S^20^). RBP eCLIP BAM files were accessed from https://www.encodeproject.org/. Raw and analyzed data for all sequencing experiments have been deposited at the GEO (https://www.ncbi.nlm.nih.gov/geo/) under accession number GSE166155 (ref. ^63^).

## Code availability

The source code of KARR-seq is freely available at https://github.com/ouyang-lab/KARR-seq (ref. ^64^).
