# Supplementary material for: KARR-seq reveals cellular higher-order RNA structures and RNA–RNA interactions
Source: Nat Biotechnol. 2024 Jan 18;42(12):1909–20. doi: 10.1038/s41587-023-02109-8 (PMC11255127; doi:10.1038/s41587-023-02109-8)
Supplement: Supplementary file 2 — Reporting Summary [file 41587_2023_2109_MOESM2_ESM.pdf]

nature research

Corresponding author(s): Zhengqing Ouyang, Chuan He

Reporting Summary

Nature Research wishes to improve the reproducibility of the work that we publish. This form provides structure for consistency and transparency in reporting. For further information on Nature Research policies, see Authors & Referees and the Editorial Policy Checklist.

Statistical parameters

When statistical analyses are reported, confirm that the following items are present in the relevant location (e.g. figure legend, table legend, main text, or Methods section).

n/a	Confirmed	
☐	☒	The exact sample size (*n*) for each experimental group/condition, given as a discrete number and unit of measurement
☐	☒	An indication of whether measurements were taken from distinct samples or whether the same sample was measured repeatedly
☐	☒	The statistical test(s) used AND whether they are one- or two-sided<br>*Only common tests should be described solely by name; describe more complex techniques in the Methods section.*
☒	☐	A description of all covariates tested
☒	☐	A description of any assumptions or corrections, such as tests of normality and adjustment for multiple comparisons
☐	☒	A full description of the statistics including central tendency (e.g. means) or other basic estimates (e.g. regression coefficient) AND variation (e.g. standard deviation) or associated estimates of uncertainty (e.g. confidence intervals)
☐	☒	For null hypothesis testing, the test statistic (e.g. *F*, *t*, *r*) with confidence intervals, effect sizes, degrees of freedom and *P* value noted<br>*Give P values as exact values whenever suitable.*
☒	☐	For Bayesian analysis, information on the choice of priors and Markov chain Monte Carlo settings
☒	☐	For hierarchical and complex designs, identification of the appropriate level for tests and full reporting of outcomes
☐	☒	Estimates of effect sizes (e.g. Cohen's *d*, Pearson's *r*), indicating how they were calculated
☐	☒	Clearly defined error bars<br>*State explicitly what error bars represent (e.g. SD, SE, CI)*

Our web collection on statistics for biologists may be useful.

Software and code

Policy information about availability of computer code

Data collection	Next-generation sequence data was collected on Illumina platforms. Nikon TiE microscope with a CFI HP TIRF objective (100×, NA 1.49, Nikon) and an EMCCD (Andor, iXon Ultra 888). FISH probes were designed using Stellaris Probe Designer (v4.2).
Data analysis	STAR (v2.5.2a), SAMtools (v1.10), pyGenomeTracks (v3.5), RNAcofold (ViennaRNA v2.4.3), DESeq2 (v1.24.0), BEDtools (v2.26.0), Cutadapt (v3.5), BWA (v0.7.9a), CTK (v1.1.3), JPGM (v1.0), GSEApy (v1.0.1), Pairix (v0.3.7), SeqPrep (commit: 575507b), Python (v2.7.18 and v3.7.12), R (v3.6.3), ImageJ (1.53n) plugin ThunderSTORM (v1.3). FlowJo(10.0.7). The source code of KARR-seq is available at https://github.com/ouyang-lab/KARR-seq

For manuscripts utilizing custom algorithms or software that are central to the research but not yet described in published literature, software must be made available to editors/reviewers upon request. We strongly encourage code deposition in a community repository (e.g. GitHub). See the Nature Research guidelines for submitting code & software for further information.

Data

Policy information about availability of data

All manuscripts must include a data availability statement. This statement should provide the following information, where applicable:

- Accession codes, unique identifiers, or web links for publicly available datasets
- A list of figures that have associated raw data
- A description of any restrictions on data availability

Raw and analyzed data for all sequencing experiments have been deposited at the GEO (https://www.ncbi.nlm.nih.gov/geo/) under accession number GSE166155. Public sequencing data used in this study are acquired from GEO with the accession number as the follows: GSE127188 (RIC-seq, HeLa); GSE74353 (PARIS, HEK293T); GSE138058 (G3BP1-APEX-seq); GSE121952 (ribosome profiling). cryoEM structures were acquired from Protein Data Bank (accession codes: 7QXB for TERC, 6QX9 for U3, 4V6X for 18S and 28S). RBP eCLIP BAM files were accessed from: www.ENCODEproject.org.

Field-specific reporting

Please select the best fit for your research. If you are not sure, read the appropriate sections before making your selection.

☒ Life sciences ☐ Behavioural & social sciences ☐ Ecological, evolutionary & environmental sciences

For a reference copy of the document with all sections, see nature.com/authors/policies/ReportingSummary-flat.pdf

Life sciences study design

All studies must disclose on these points even when the disclosure is negative.

Sample size	No statistical methods were used to predetermine sample size. For both sequencing data and biochemical assays, experiments were performed in duplicates or triplicates, which is common in similar studies (Xue et al., Nature 2020; Lu et al., Cell 2016).
Data exclusions	No data was excluded from analysis.
Replication	Results were confirmed in two or three biological replicates for each experiment unless otherwise stated. All attempts to replicate data were successful.
Randomization	Randomization is not applicable because all experiments were performed in cell lines. Comparison were performed with control experiments to eliminate the effect of confounders.
Blinding	Blinding is not relevant to this study because no group allocation was involved. Samples were treated and analyzed using the same protocol.

Reporting for specific materials, systems and methods

Materials & experimental systems

n/a	Involved in the study
☒	☐ Unique biological materials
☐	☒ Antibodies
☐	☒ Eukaryotic cell lines
☒	☐ Palaeontology
☒	☐ Animals and other organisms
☒	☐ Human research participants

Methods

n/a	Involved in the study
☒	☐ ChIP-seq
☐	☒ Flow cytometry
☒	☐ MRI-based neuroimaging

Antibodies

Antibodies used

Rabbit anti-SRSF1 (Bethyl, catalog number: A302-052A); 1:1,000 dilution.
Rabbit anti-Histone H3 (Cell Signaling Technologies, catalog number: 9717, clone number: 3H1); 1:1,000 dilution.
Rabbit anti-β–tubulin (Cell Signaling Technologies, catalog number: 2146); 1:1,000 dilution.
Rabbit anti-U1-70K (Abcam, catalog number: ab83306); 1:1,000 dilution.
Rabbit anti-YBX3 (GeneTex, catalog number: GTX130052); 1:1,000 dilution.
Mouse anti-GAPDH (Proteintech, catalog number: HRP-60004), 1:20,000 dilution.
Anti-rabbit IgG, HRP-linked (Cell Signaling Technologies, catalog number: 7074), 1:2,500 dilution.

Validation	Validation of all primary antibodies for Western blot using human lysates are available on the manufacturers' websites. Links are as the follows:<br>Rabbit anti-SRSF1: https://www.fortislife.com/products/primary-antibodies/rabbit-anti-asf-antibody/BETHYL-A302-052<br>Rabbit anti-Histone H3: https://www.cellsignal.com/products/primary-antibodies/histone-h3-3h1-rabbit-mab/9717<br>Rabbit anti-β–tubulin: https://www.cellsignal.com/products/primary-antibodies/b-tubulin-antibody/2146<br>Rabbit anti-U1-70K: https://www.abcam.com/products/primary-antibodies/snrp70u1-70k-antibody-ab83306.html<br>Rabbit anti-YBX3: https://www.genetex.com/Product/Detail/CSDA-antibody/GTX130052<br>Mouse anti-GAPDH: https://www.ptglab.com/products/GAPDH-Antibody-60004-1-Ig.htm

Eukaryotic cell lines

Policy information about cell lines

Cell line source(s)	A549 (ATCC, catalog number: CCL-185), HEp-2 (ATCC, catalog number: CCL-23), Vero CCL81 (ATCC, catalog number: CRLCCL81), HEK293T (ATCC, catalog number: CRL11268), HepG2 (ATCC, catalog number: HB8065), K562 (ATCC, catalog number: CCL243), Drosophila S2 (Thermo Fisher, catalog number: R69007), Murine embryonic stem cells F123 (Bing Ren lab, UCSD)
Authentication	None of the cell lines used were authenticated.
Mycoplasma contamination	All cell lines used in this study were tested negative of mycoplasma contamination.
Commonly misidentified lines<br>(See ICLAC register)	No commonly misidentified line was used.

Flow Cytometry

Plots

Confirm that:

☒ The axis labels state the marker and fluorochrome used (e.g. CD4-FITC).

☒ The axis scales are clearly visible. Include numbers along axes only for bottom left plot of group (a 'group' is an analysis of identical markers).

☒ All plots are contour plots with outliers or pseudocolor plots.

☒ A numerical value for number of cells or percentage (with statistics) is provided.

Methodology

Sample preparation	A549 cells were infected by rVSV-GFP (VSV expressing GFP) at an MOI of 0.1. Mock-infected A549 cells were used as controls. After infection, cells were trypsinized and fixed by 4% paraformaldehyde solution. Cells with the concentration of 1 – 10 million cells/mL were used for each assay. Cells were suspended in sample buffer (PBS, 1mM EDTA) containing 0.2 % BSA or 10% heat-inactivated FBS. Samples were filtered by cell-strainer caps before analysis.
Instrument	Attune NxT Flow Cytometer (Thermo Fisher)
Software	FlowJo (10.0.7)
Cell population abundance	10,000 to 50,000 cells were collected according for each assay.
Gating strategy	The first step in gating is to use the counter map of FSC/SSC to exclude cell debris and dead cells. The population of control group and sample group were gated by fluorescence either using two parameter density plot (Fluorescence vs SSC) or single parameter histograms (Fluorescence vs intensity). The mock-infected cells (GFP negative) were used for gating. The percent of GFP positive cells were calculated. Results are the averages from three independent experiments and are expressed as mean ± standard errors.

☒ Tick this box to confirm that a figure exemplifying the gating strategy is provided in the Supplementary Information.
