# Supplementary material for: KARR-seq reveals cellular higher-order RNA structures and RNA–RNA interactions
Source: Nat Biotechnol. Author manuscript; Available in PMC 2025 Dec 1. (PMC11255127; doi:10.1038/s41587-023-02109-8)
Supplement: Supplementary file 1 [file NIHMS1966009-supplement-Sup_Figures.pdf]

nature biotechnology

Article https://doi.org/10.1038/s41587-023-02109-8

KARR-seq reveals cellular higher-order RNA structures and RNA–RNA interactions

In the format provided by the authors and unedited

Table of Contents

Supplementary Figures

Supplementary Figure 1. Quality controls for KARR-seq.

Supplementary Figure 2. Evaluating the background level of KARR-seq ligation.

Supplementary Figure 3. The performance of different dendrimers in KARR-seq.

Supplementary Figure 4. KARR-seq reveals RNA tertiary structures.

Supplementary Figure 5. Transcriptome-wide comparison between KARR-seq, PARIS, and RIC-seq.

Supplementary Figure 6. Comparing KARR-seq, PARIS, and RIC-seq using individual transcripts.

Supplementary Figure 7. KARR-seq using purified cell nucleus.

Supplementary Figure 8. Distinct higher-order mRNA structures in the cell nucleus.

Supplementary Figure 9. Comparison of KARR-seq performed under strong, weak, and no crosslinking conditions in K562 cells.

Supplementary Figure 10. Using folding index to describe RNA foldability.

Supplementary Figure 11. The interplay between RBP and RNA high-order structures.

Supplementary Figure 12. Translation suppresses higher-order mRNA structures.

Supplementary Figure 13. KARR-seq identified diverse intermolecular RNA–RNA interactions.

Supplementary Figure 14. RNA–RNA interactions between RSV/VSV and host RNAs.

Supplementary Figure 15. Gating Strategy to quantify GFP-positive A549 cells.

Supplementary Figure 16. Uncropped scans for Western blots.

Supplementary Protocol

Supplementary Figures

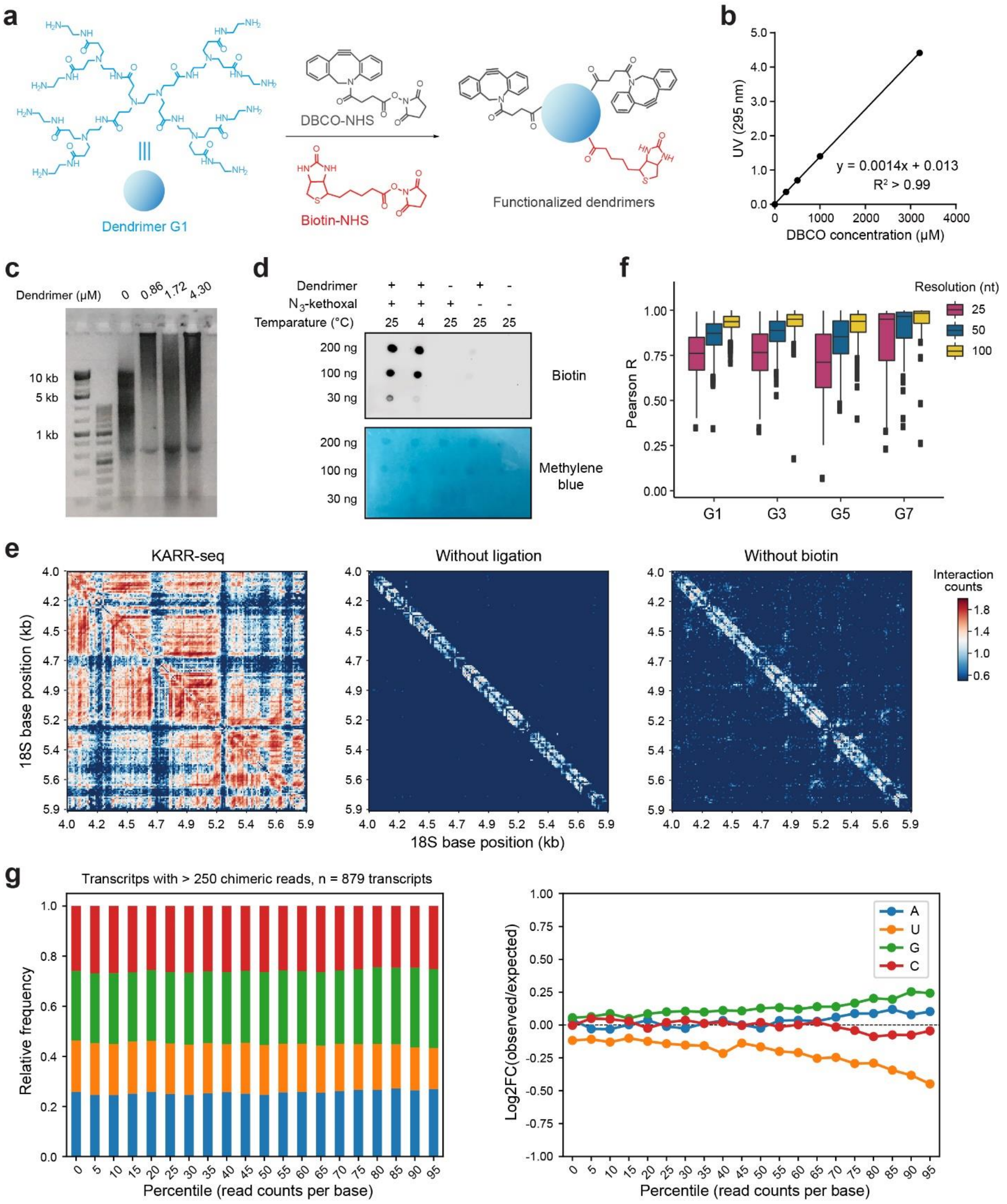

Supplementary Figure 1. Quality controls for KARR-seq. (**a**) The scheme illustrating the synthesis of biotin and DBCO-modified dendrimers, using G1 as an example. The detailed synthetic procedure is included in Methods. (**b**) The calibration curve for quantifying DBCO-containing dendrimer, using DBCO-NHS as the standard. The characteristic UV adsorption of DBCO-NHS at 295 nm was plotted across

different concentrations. R^2 denotes the Pearson correlation coefficient. (**c**) Gel electrophoresis analysis of crosslinked and uncrosslinked RNA from mESCs. The first and the second lanes of the gel are DNA size markers. (**d**) Dot blot analysis of the crosslinked RNA. Dendrimer and/or N_3-kethoxal treatment were omitted in the negative control samples. Images are representatives of two independent repeats. (**e**) KARR-seq interaction maps for 18S in mESCs. KARR-seq performed without proximity ligation or using biotin-free dendrimers detected minimal contact frequency. (**f**) Pearson correlation coefficients between KARR-seq interaction maps generated from two replicates in mESCs. Transcripts (TPM > 1.0) detected in both replicate samples were included for analysis. For each transcript, Pearson correlation was analyzed at different resolutions. The distribution of all Pearson correlation coefficients was plotted. In (**e**) and (**f**), KARR-seq was performed in two biological replicates. (**g**) Left: the frequency of each nucleobase at positions covered by different numbers of KARR-seq chimeric reads. Right: relative enrichment of each nucleobase at positions covered by different numbers of KARR-seq chimeric reads. Transcripts with more than 250 chimeric reads were analyzed (n = 879 transcripts) to ensure enough chimeric read coverage across the transcript.

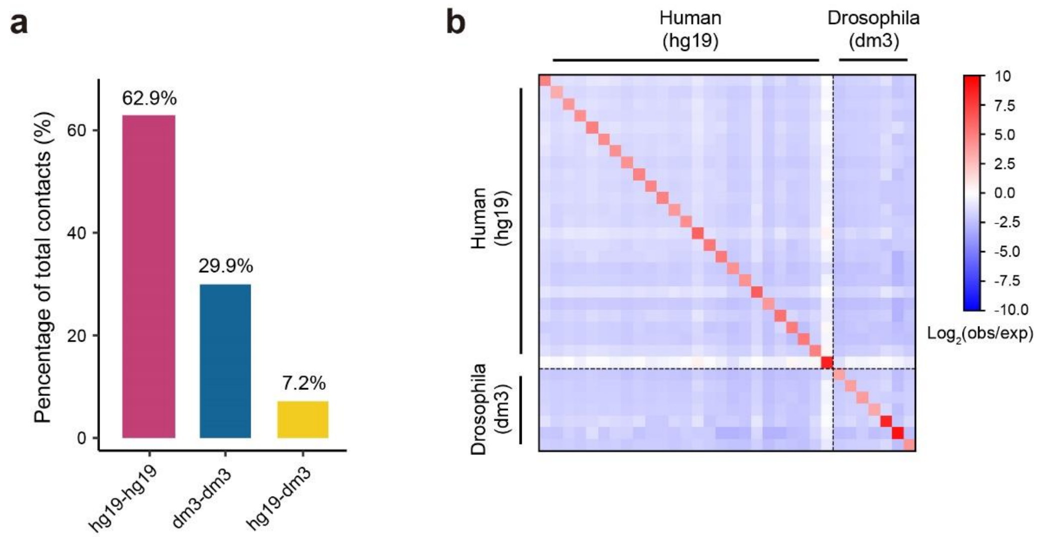

Supplementary Figure 2. Evaluating level of random ligation in KARR-seq. (**a**) The percentage of chimeric reads between different reference genomes when KARR-seq was performed using a 1:1 mixture of K562 and S2 cells. (**b**) Heat map showing observed RNA–RNA interactions across hg19 and dm3 genomes when KARR-seq was performed using a 1:1 mixture of K562 and S2 cells. No significant interspecies interaction was observed. KARR-seq was performed in two biological replicates.

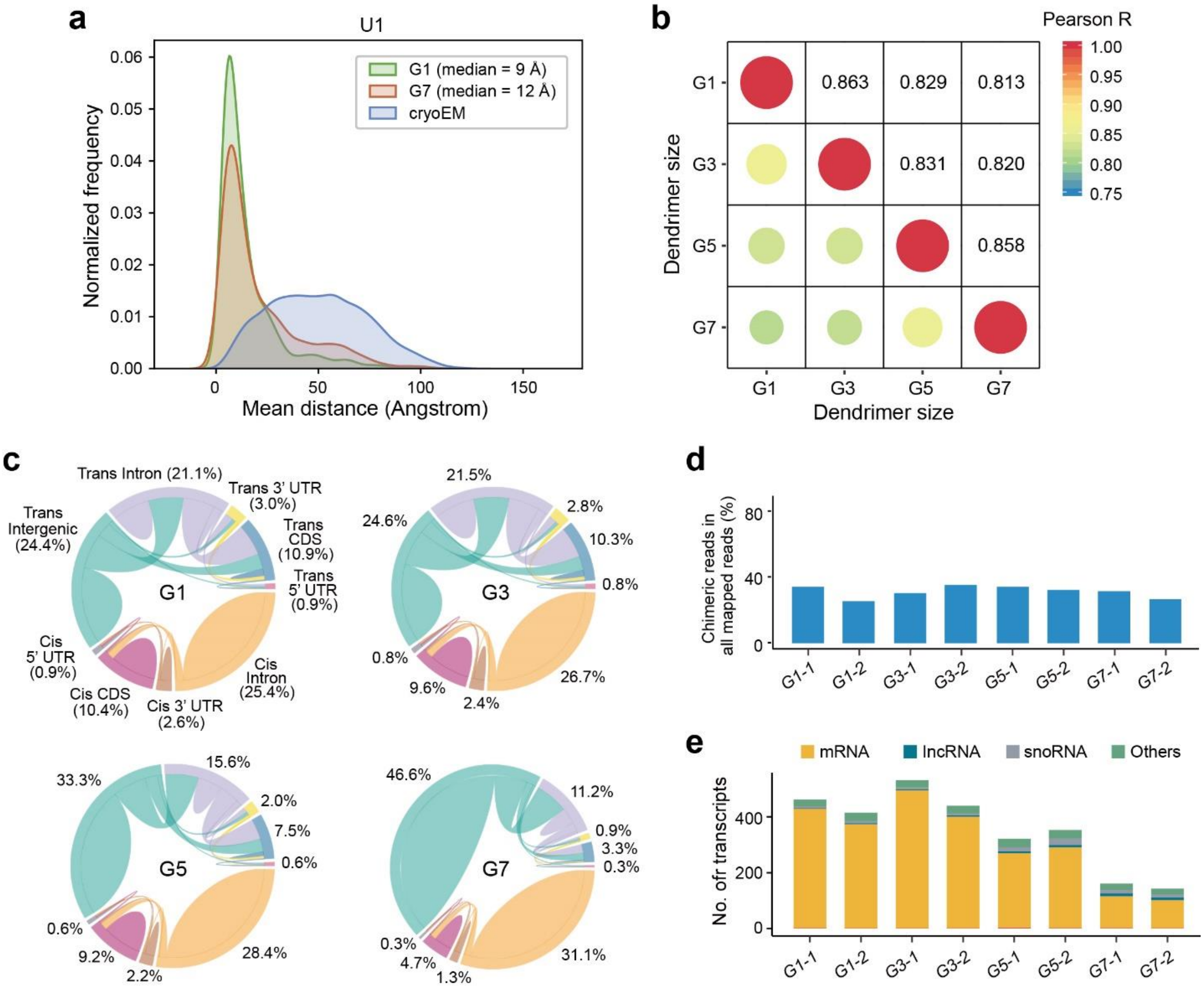

Supplementary Figure 3. The performance of different dendrimers in KARR-seq. (**a**) Physical distances between interacting fragments of U1 in K562 cells, measured by KARR-seq data generated using G1 and G7. The physical distances were measured using the cryoEM structures of U1. The actual physical distance distribution in the cryoEM structure is shown in blue for comparison. (**b**) Pearson correlation between KARR-seq data generated by different dendrimers in mESCs. (**c**) Circos plots showing RNA–RNA interaction landscapes in mESCs revealed by KARR-seq using denoted dendrimers. The width of the link between two RNA categories indicates the percentage of chimeric reads taken by interactions between these categories. (**d**) The ratio of chimeric reads over all uniquely mapped KARR-seq reads when using different dendrimers. (**e**) The number of transcripts with valid RNA–RNA interactions captured by different dendrimers. KARR-seq was performed in two biological replicates.

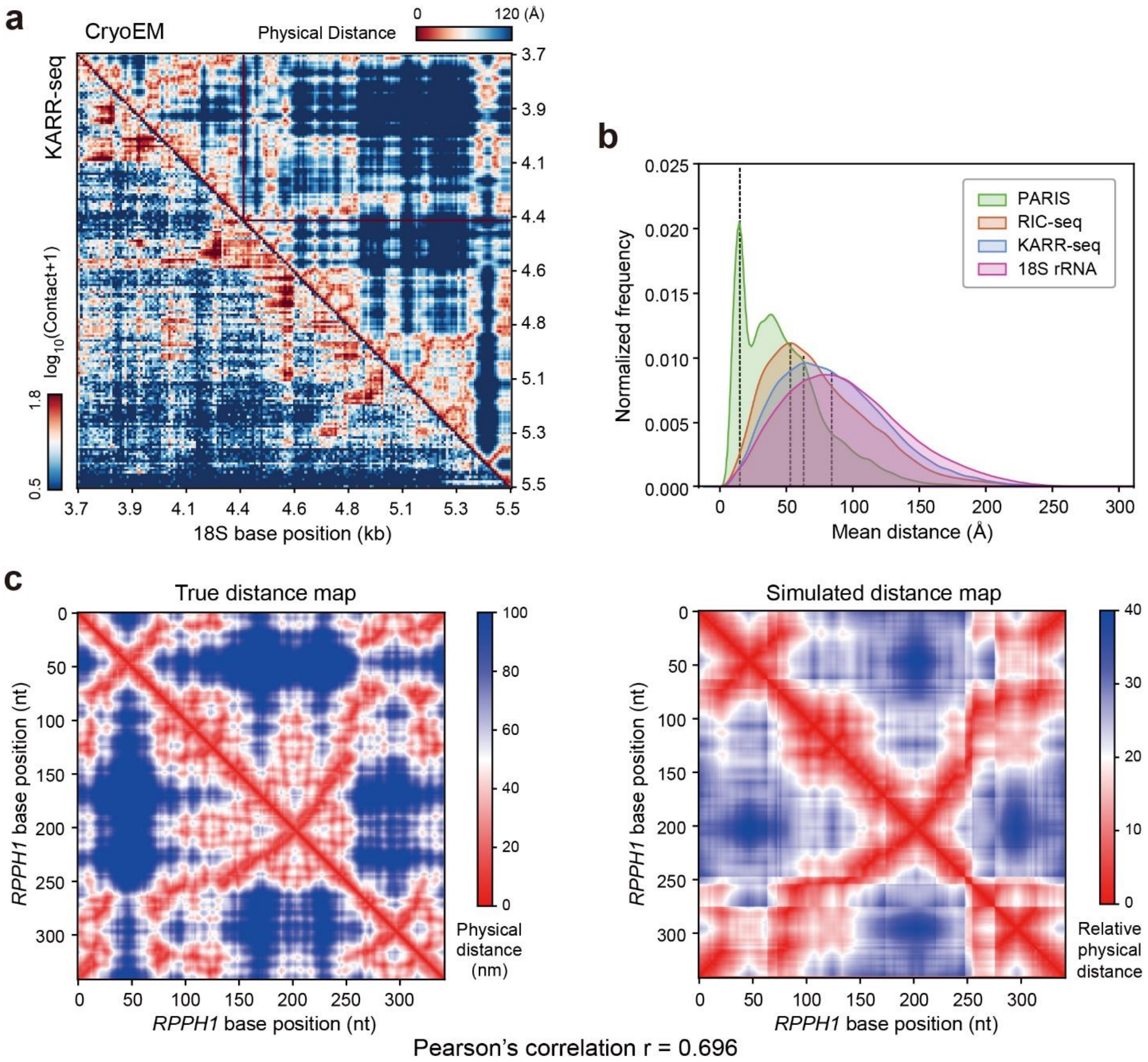

Supplementary Figure 4. KARR-seq reveals RNA tertiary structures. (**a**) The comparison between KARR-seq contact frequency and physical distances within 18S rRNA. Bottom left: the map of KARR-seq contact frequency for 18S rRNA in K562 cells. Top right: the physical distance map of 18S taken from the CryoEM structure of the ribosome. (**b**) Physical distances between the two interacting fragments mapped to 18S from KARR-seq (blue), PARIS (green), and RIC-seq (brown) data. Two arms of each chimeric read were first aligned to the 18S CryoEM map. The physical distances between the mid-point of the two arms were then measured and plotted. The actual physical distance distribution in the 18S CryoEM map was shown in purple for comparison. (**c**) Left: the true physical distance map of *RPPH1* transcript revealed by cryoEM structure (PDB: 6AHR). Right: The relative physical distance map by simulation. The Pearson's correlation coefficient is 0.696. Note that the simulation only reveals relative but not absolute physical distances. KARR-seq was performed in two biological replicates.

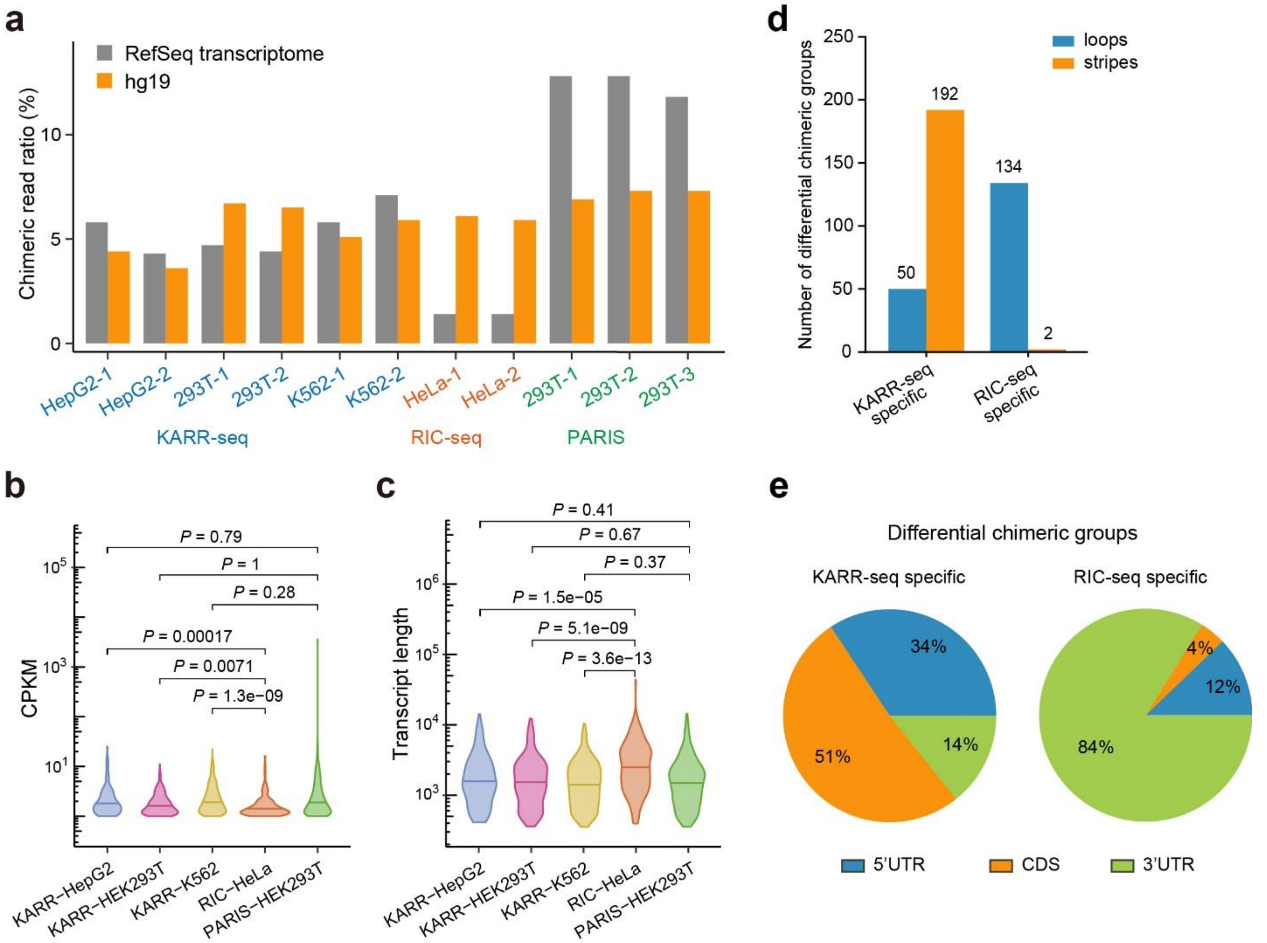

Supplementary Figure 5. Transcriptome-wide comparison between KARR-seq, PARIS, and RIC-seq. (**a**) The ratio of chimeric reads for KARR-seq, PARIS, and RIC-seq when data were mapped to RefSeq transcriptome and hg19. Data from individual replicates were shown separately. (**b**) The abundance (RPKM) of chimeric reads for mRNAs with valid intramolecular interactions detected by KARR-seq, PARIS, and RIC-seq. (**c**) The length distribution for mRNAs with valid intramolecular interactions detected by KARR-seq, PARIS, and RIC-seq. For (b) and (c), n=132 transcripts for KARR-HepG2, n=326 transcripts for KARR-HEK293T, n=520 transcripts for KARR-K562, n=159 transcripts for RIC-HeLa, n=488 transcripts for PARIS-HEK293T. (**d**) The number of loops and stripes among KARR-seq-specific and RIC-seq-specific RNA–RNA interactions revealed by differential analysis. (**e**) The locality of KARR-seq-specific and RIC-seq-specific RNA–RNA interactions revealed by differential analysis. RIC-seq and PARIS data was acquired from GEO (RIC-seq: HeLa cells, GSE127188; PARIS: HEK293T cells, GSE74353). KARR-seq was performed in two biological replicates.

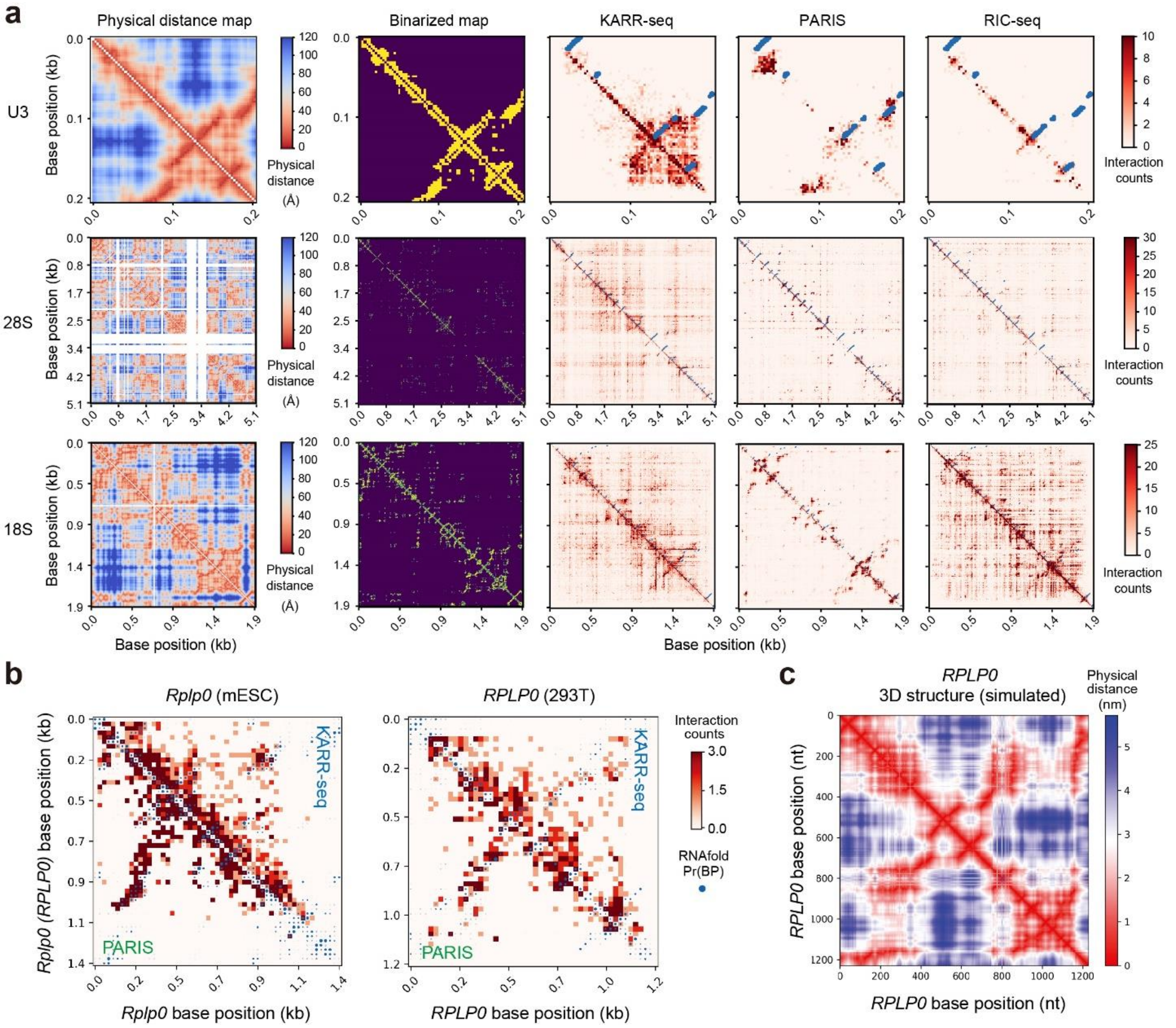

Supplementary Figure 6. Comparing KARR-seq, PARIS, and RIC-seq using individual transcripts. (**a**) The physical distance maps, binarized maps, and interactions maps revealed by KARR-seq, PARIS, and RIC-seq data for U3, 28S, and 18S. Physical distance maps were revealed by cryoEM structures acquired from Protein Data Bank (accession codes: 6QX9 for U3, 4V6X for 18S and 28S). Binarized maps were applied in the ROC–AUC analysis. (**b**) Interaction maps for *Rplp0* (*RPLP0*) mRNA in mESCs and HEK293T cells generated from KARR-seq (top right) and PARIS (bottom left) data. (**c**) The simulated physical distance map of the human *RPLP0* mRNA.

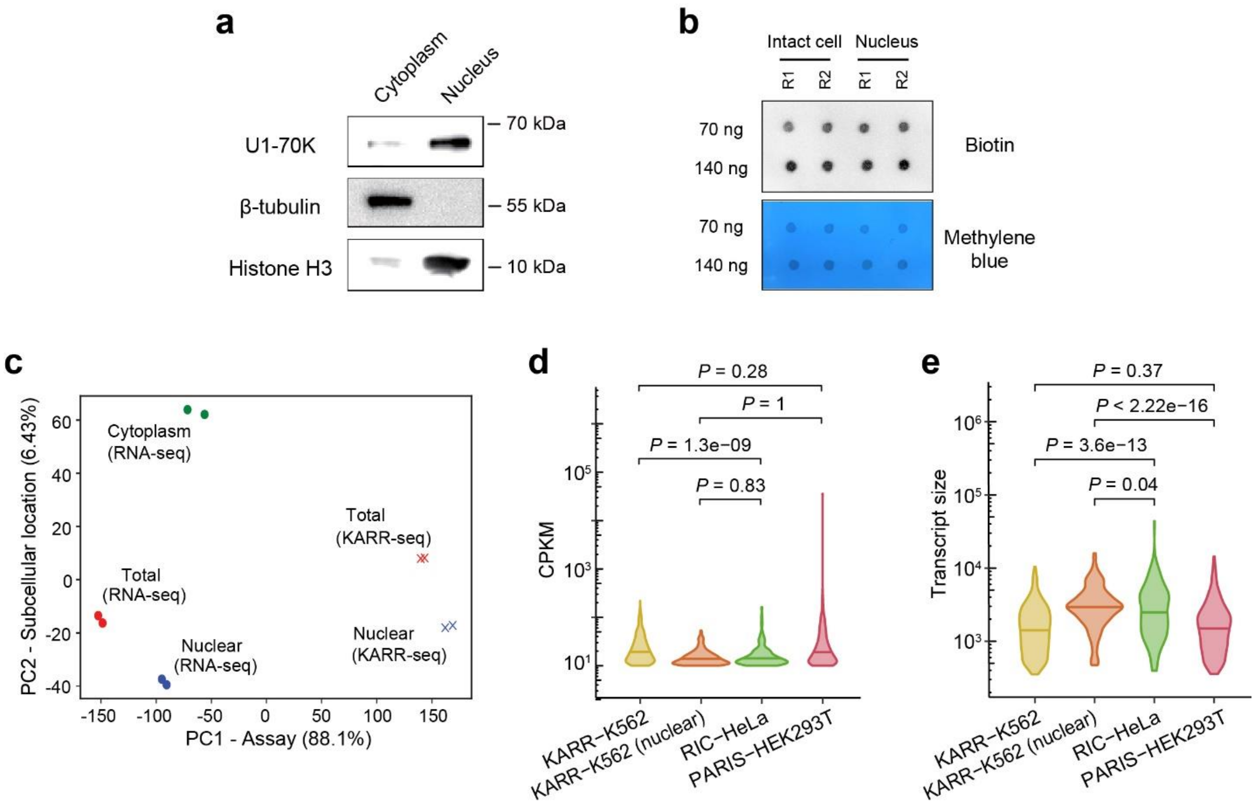

Supplementary Figure 7. KARR-seq using purified cell nucleus. (**a**) Western blotting of U1-70K, histone H3, and β-tubulin in the cytoplasmic and nuclear fractions as an indicator of successful cell fractionation in K562 cells. Images are representatives of two independent repeats. (**b**) Dot blot showing the strength of biotin signals on crosslinked total RNA and nuclear RNA that were used for KARR-seq experiments. R1 and R2 denote two different RNA samples. (**c**) PCA of KARR-seq and RNA-seq data produced from intact K562 cells (total) or K562 nuclei (nuclear). Whole-cell (total), cytoplasm, and nuclear RNA-seq results were plotted for comparison. (**d**) The abundance (RPKM) of chimeric reads for mRNAs with valid intramolecular interactions detected by PARIS, RIC-seq, and KARR-seq using intact cells or cell nuclei. (**e**) The length distribution for mRNAs with valid intramolecular interactions detected by PARIS, RIC-seq, and KARR-seq using intact cells or cell nuclei. For (**d**) and (**e**), n=520 transcripts for KARR-K562, n=354 transcripts for KARR-K562 (nuclear), n=159 transcripts for RIC-HeLa, n=488 transcripts for PARIS-HEK293T. *P* values were calculated by the Mann-Whitney test. RIC-seq and PARIS data was acquired from GEO (RIC-seq: HeLa cells, GSE127188; PARIS: HEK293T cells, GSE74353). KARR-seq was performed in two biological replicates.

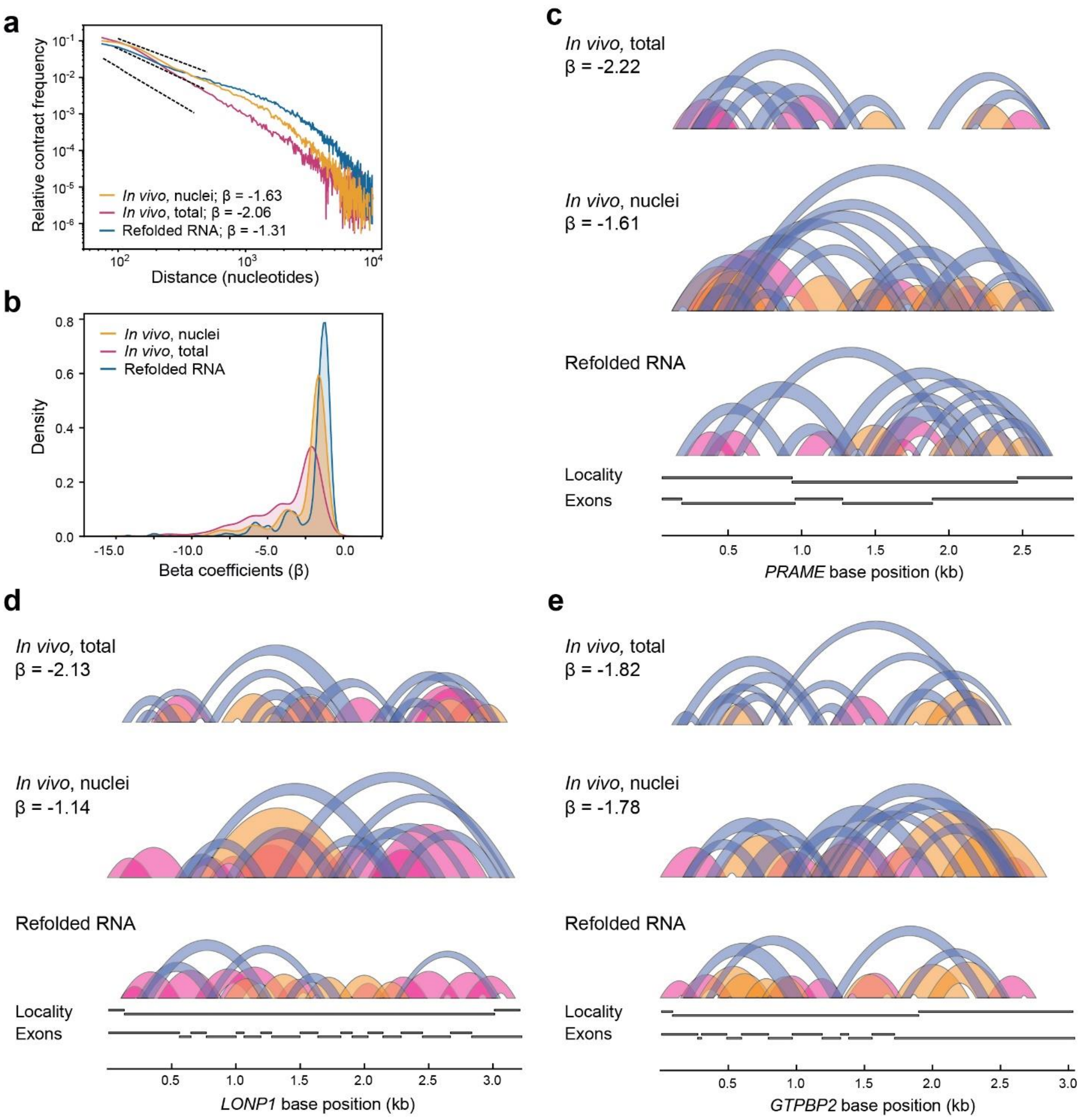

Supplementary Figure 8. Distinct higher-order mRNA structures in the cell nucleus. (**a**) The relationship between RNA coordinate distance and relative contact frequency in intact K562 cells, K562 nuclei, and re-folded K562 RNA. Beta coefficients (β) were calculated as the slope of the line at the 100–1,000 nucleotides region. (**b**) The distribution of beta coefficients for individual mRNAs in intact K562 cells, K562 nuclei, and re-folded K562 RNA. (**c**)–(**e**) KARR-seq arc groups for *PRAME*, *LONP1*, and *GTPBP2* mRNAs from re-folded K562 RNA, intact K562 cells (*in vivo*, total), and K562 nuclei (*in vivo*, nuclei). Beta coefficients were noted for each transcript from intact cells and purified nuclei. For (**a**) and (**b**), transcripts that were presented in all three groups were included in the analysis.

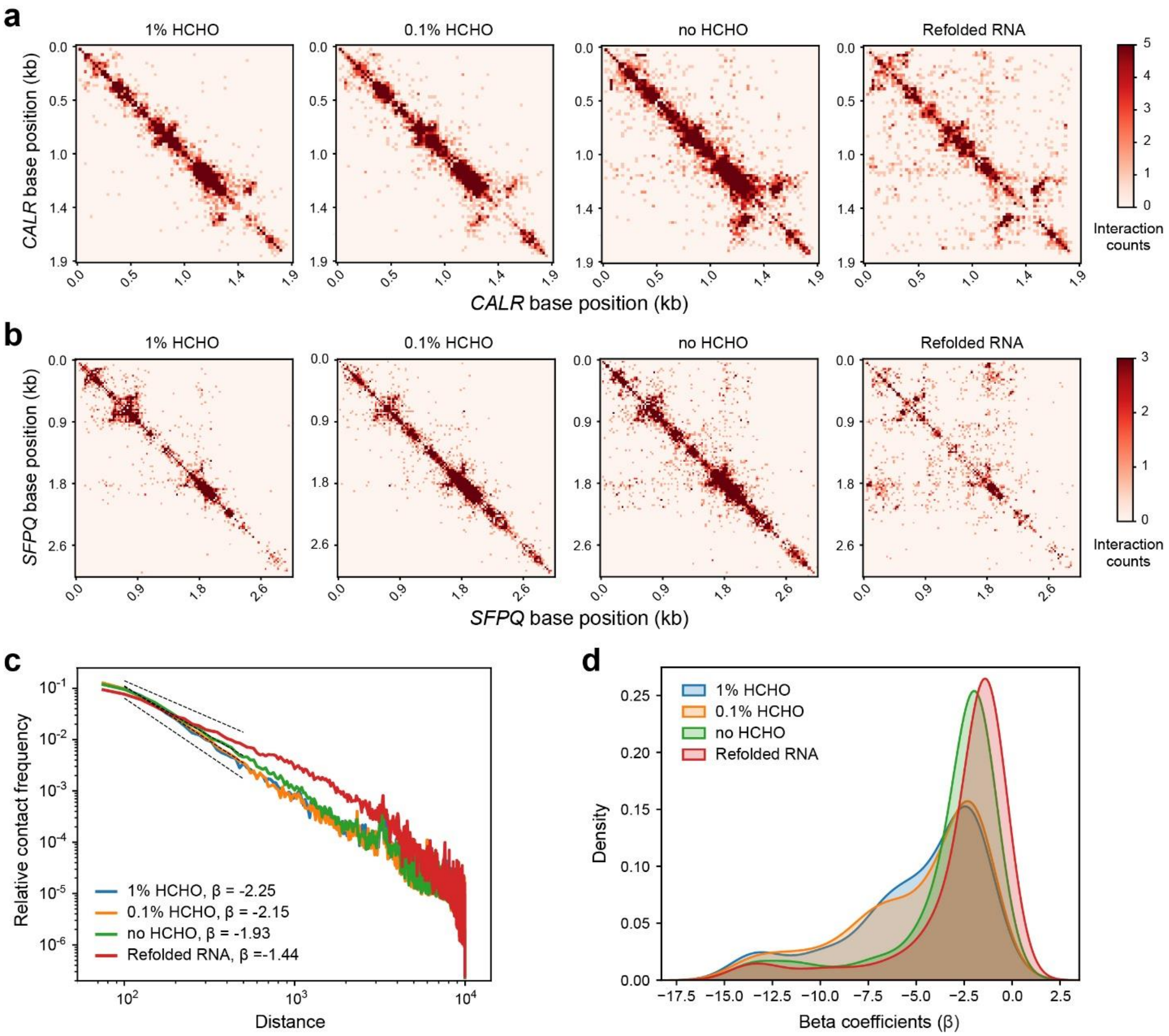

Supplementary Figure 9. The effect of formaldehyde crosslinking in detecting RNA–RNA interactions by KARR-seq. (**a**)–(**b**) KARR-seq interaction maps for *CALR* and *SFPQ* mRNAs under denoted conditions in K562 cells. (**c**) The relationship between RNA coordinate distance and relative contact frequency under denoted conditions in K562 cells. Beta coefficients (β) were calculated as the slope of the line at 100–1,000 nucleotides region. (**d**) The distribution of beta coefficients for individual mRNA transcripts under denoted conditions in K562 cells. For (**c**) and (**d**), transcripts that were presented in all four groups were included in the analysis.

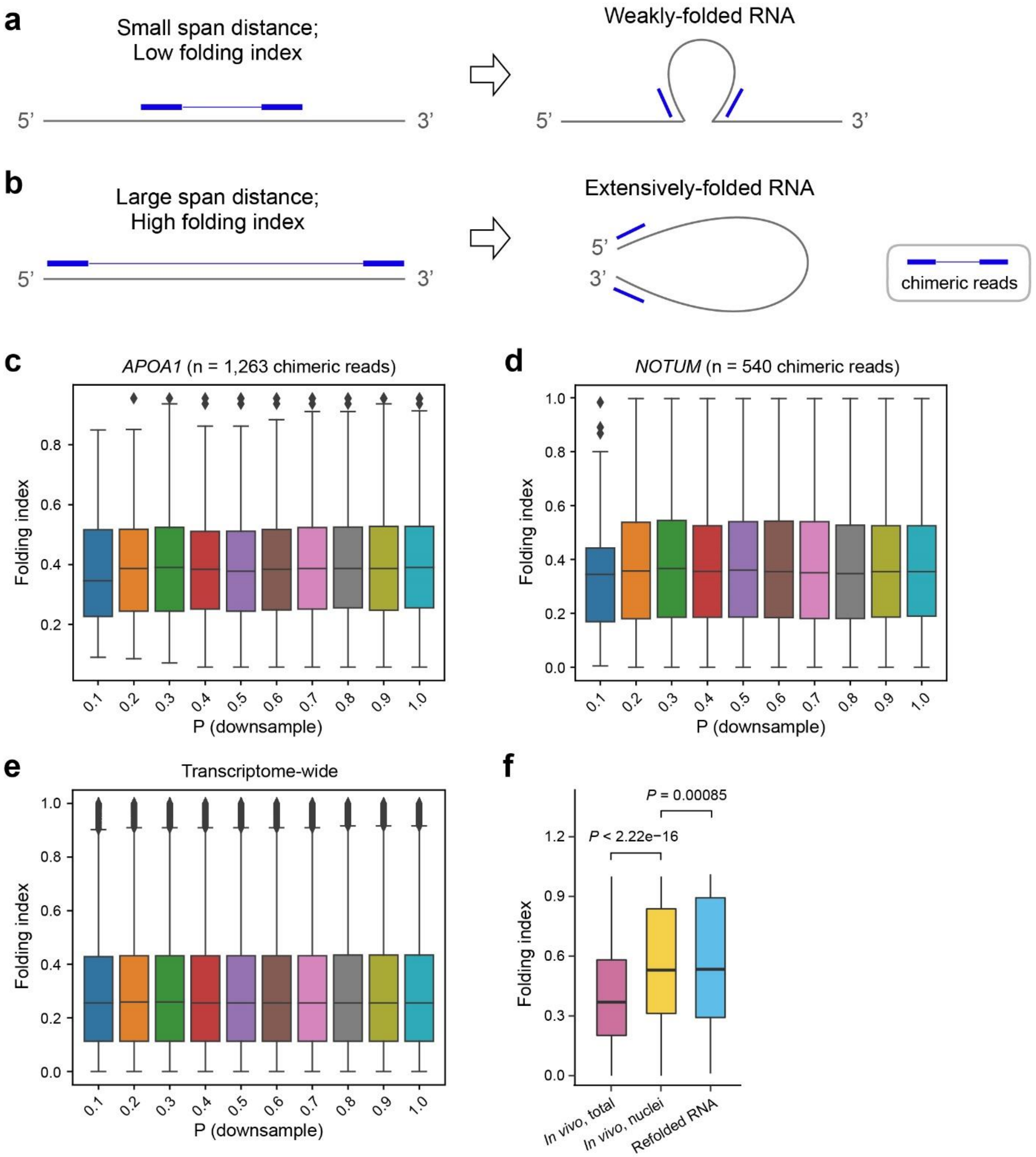

Supplementary Figure 10. Using folding index to describe RNA foldability. (**a**)–(**b**) The schematic illustration of folding index. In (a), the left and the right arms of a chimeric read were mapped to two closely located regions. This corresponds to a small span distance and a low folding index, indicating that the transcript is weakly folded. In (b), the left and right arms of the chimeric reads were mapped to two distant regions, which correspond to a large span distance, a high folding index, and extensive folding. (**c**)–(**d**) The folding index distribution for *APOA1* and *NOTUM* in HepG2 cells when the numbers of chimeric reads mapped to them (1,263 for *APOA1* and 540 for *NOTUM*) were downsized to as few as 10%. (**e**) The transcriptome-wide distribution of folding index in HepG2 cells when chimeric reads were downsized to as few as 10%. 155,714 chimeric reads are included in P=1.0. (**f**) Folding index for mRNA in intact K562 cells, K562 nuclei, and re-folded K562 RNA. n refers to the number of chimeric read level folding index.

n=376,853 for in vivo total, n=337,868 for in vivo nuclei, n=277,014 for refolded RNA. *P* values were calculated using the two-sided Mann-Whitney test. KARR-seq was performed in two biological replicates. In box plots shown in (c)–(f), the lower and the upper bounds denoted the 25th and 75th percentile, respectively. The minima denote the lower bound - 1.5 ×IQR (interquartile range). The maxima denote the upper bound + 1.5 ×IQR.

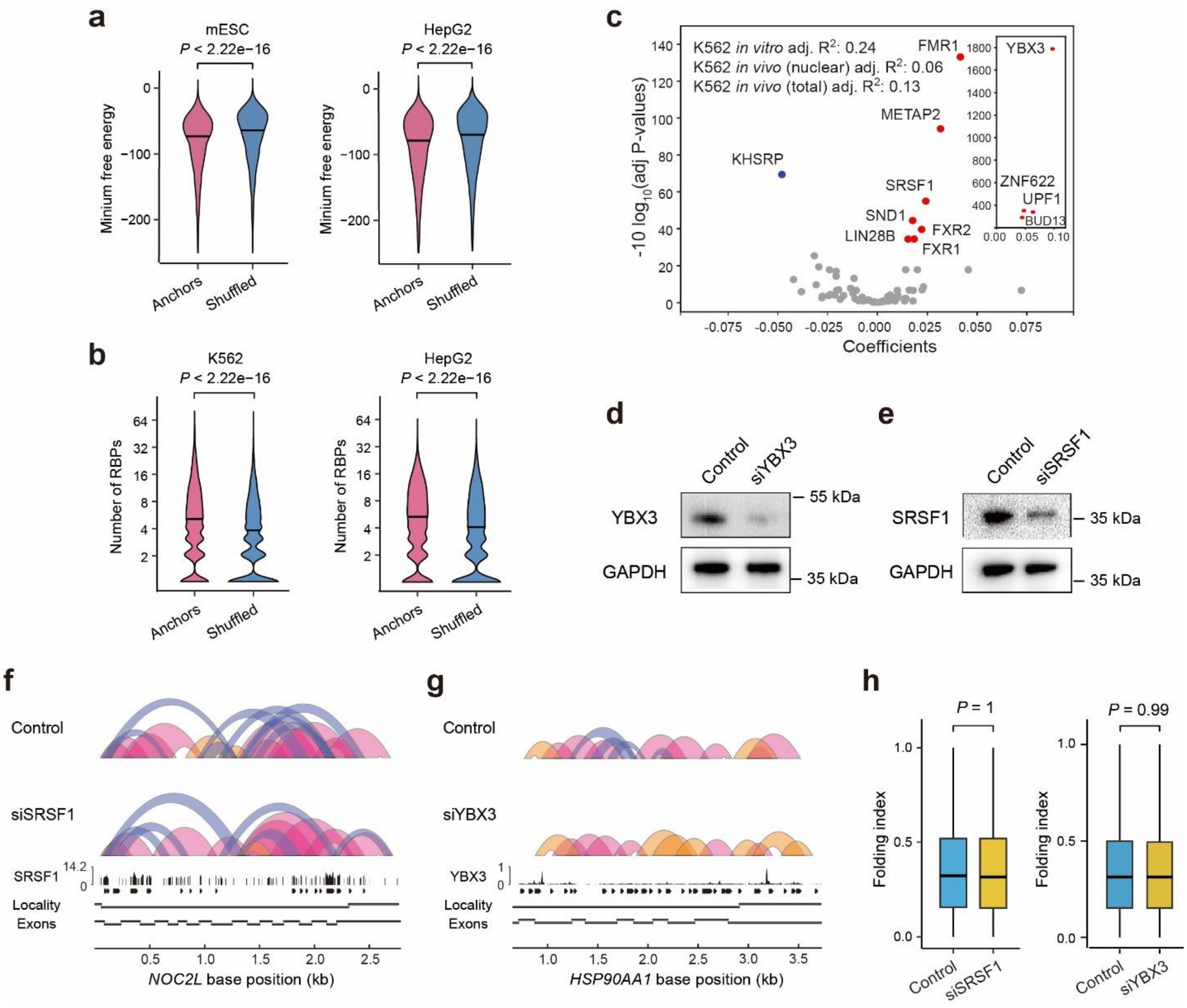

Supplementary Figure 11. The interplay between RBP and high-order RNA structures. (**a**) The distribution of minimal free energy at defined interaction anchors and randomly shuffled regions in mESCs and HepG2 cells. n=7,518 regions for mESC (anchors and shuffled), n=12,781 regions for HepG2 (anchors and shuffled). Interaction anchors were defined as regions with maximal KARR-seq chimeric reads coverage within the two interacting fragments using refolded RNA. *P* values were calculated by the one-sided Mann-Whitney test. (**b**) The number of RBP-binding sites at interaction anchors and randomly shuffled mRNA regions in HepG2 and K562 cells. n=39,167 regions for K562 anchors, n=39,090 regions for K562 shuffled, n=17,916 regions for HepG2 anchors, n=17,944 regions for HepG2 shuffled. *P* values were calculated by the one-sided Mann-Whitney test. (**c**) Linear regression fit between the mRNA folding index and the binding of individual RBP in K562 cells. The x-axis shows the correlation coefficient for each RBP, with higher coefficients denoting a stronger association between RBP and RNA–RNA interactions. Proteins that possess significant positive or negative correlations with RNA–RNA interactions were colored in red and blue, respectively. One-sided F-Test was performed for the ordinary least squares regression. Multiple comparisons were done using the Benjamini-Hochberg procedure. (**d**)–(**e**) Western blots showing the protein levels for YBX3 (**c**) and SRSF1 (**d**) in the knockdown K562 cells and the corresponding control cells. Images are representatives of two independent replicates. (**f**) KARR-seq arc groups for the *NOCL2* mRNA in *SRSF1* knockdown and the corresponding control K562 cells. Folding index: 0.477 for Control, 0.474 for siSRSF1. (**g**) KARR-seq arc groups for the *HSP90AA1* mRNA in *YBX3* knockdown and the corresponding control K562 cells. Folding index: 0.265 for Control, 0.279 for siYBX3.

(**h**) mRNA folding index in *SRSF1* knockdown, *YBX3* knockdown, and the corresponding control K562 cells. n refers to the number of chimeric read level folding index. n=1,341,521 in control for siSRSF1, n=1,177,772 in siSRSF1, n=739,827 in control for siYBX3, n=752,662 for siYBX3. The lower and the upper bounds denoted the 25th and 75th percentile, respectively. The minima denote the lower bound - 1.5 ×IQR (interquartile range). The maxima denote the upper bound + 1.5 ×IQR. *P* values were calculated by Wilcoxon Rank-Sum. KARR-seq was performed in two biological replicates.

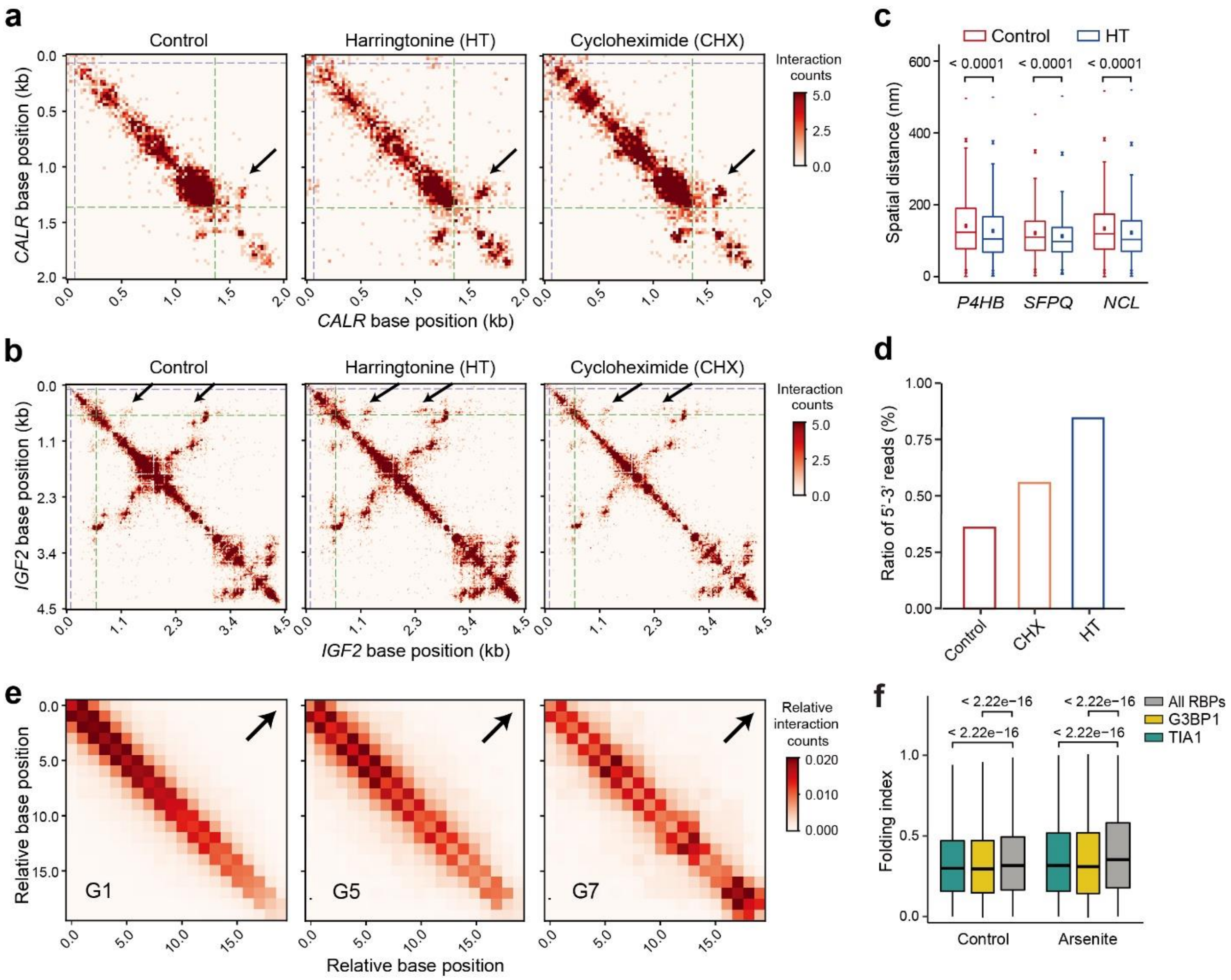

Supplementary Figure 12. Translation suppresses higher-order mRNA structures. (**a**)–(**b**) KARR-seq interaction maps for *CALR* and *IGF2* mRNAs in control HepG2 cells and HepG2 cells treated with translation inhibitors. Arrows point out gained intramolecular interactions upon translation inhibition. Dashed lines denote the boundaries between CDS and UTRs. (**c**) The spatial distance between the 5' and the 3' ends of *P4HB*, *SFPQ*, and *NCL* revealed by RNA FISH in control HepG2 cells and HepG2 cells treated with harringtonine. n refers to the number of spatial distances. n=7,484 for *P4HB* control, n=8,343 for *P4HB* HT, n=1,989 for *SFPQ* control, n=2,235 for *SFPQ* HT, n=4,453 for *NCL* control, n=3,704 for *NCL* HT. *P* values were calculated using unpaired Student's t-test. (**d**) The portion of mRNA 5'–3' chimeric reads among all chimeric reads in HepG2 cells under denoted conditions. (**e**) The meta KARR-seq interaction maps showing the relative level of intramolecular mRNA interactions across the transcriptome. Data produced by G1, G5, and G7 were plotted respectively. The arrows point to the top right corner of the maps, where 5'–3' interactions should be expected. (**f**) The folding index for mRNA binding targets of G3BP1, TIA1, and other RBPs in control and arsenite-treated K562 cells. n refers to the number of chimeric read level folding index. n=440,484 for control (all RBPs), n=433,845 for control (G3BP1), n=307,735 for control (TIA1), n=242,268 for arsenite (all RBPs), n=202,553 for arsenite (G3BP1), n=142,994 for arsenite (TIA1). *P* values were calculated by the Mann-Whitney test. KARR-seq was performed in two biological replicates.

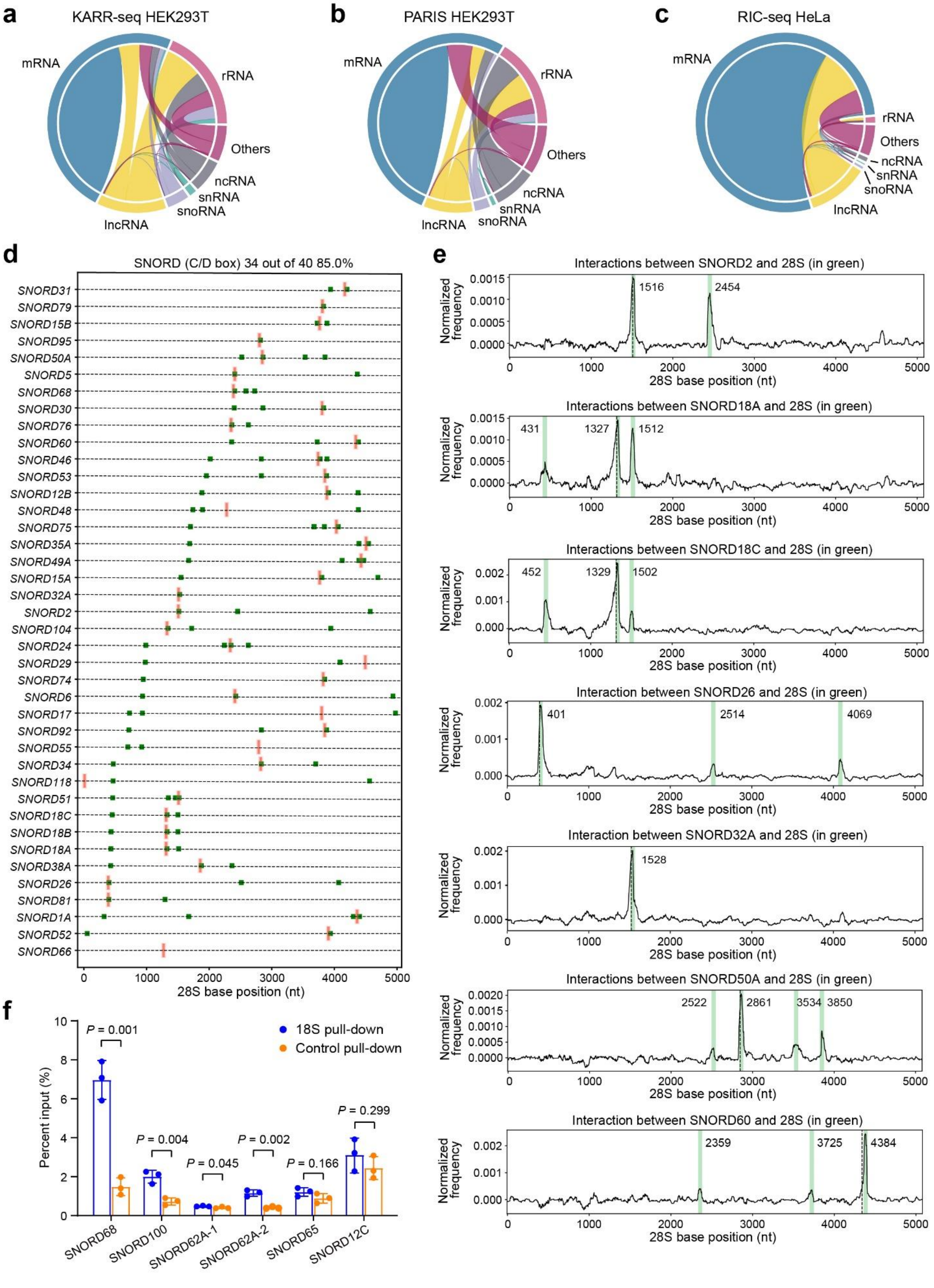
a
KARR-seq HEK293T
b
PARIS HEK293T
c
RIC-seq HeLa
mRNA
rRNA
Others
ncRNA
snRNA
snoRNA
lncRNA
d
SNORD (C/D box) 34 out of 40 85.0%
28S base position (nt)
e
Interactions between SNORD2 and 28S (in green)
Interactions between SNORD18A and 28S (in green)
Interactions between SNORD18C and 28S (in green)
Interaction between SNORD26 and 28S (in green)
Interaction between SNORD32A and 28S (in green)
Interactions between SNORD50A and 28S (in green)
Interaction between SNORD60 and 28S (in green)
Normalized frequency
28S base position (nt)
f
18S pull-down
Control pull-down
Percent input (%)
P = 0.001
P = 0.004
P = 0.045
P = 0.002
P = 0.166
P = 0.299
SNORD68
SNORD100
SNORD62A-1
SNORD62A-2
SNORD65
SNORD12C

Supplementary Figure 13. KARR-seq identified diverse intermolecular RNA–RNA interactions. (**a**)–(**c**) The landscape of intermolecular RNA–RNA interactions revealed by KARR-seq (a), PARIS (b), and RIC-seq (c). The width of the link between two RNA categories denotes the relative abundance of chimeric reads taken by interactions between these two categories. mRNA–rRNA interactions, which are primarily a result of translation, were excluded from the analysis. (**d**) Interactions between C/D box snoRNA and 28S in K562 cells. Previously identified interaction sites were shown in pink. Interaction sites identified by KARR-seq were shown in green. (**e**) Snapshots of KARR-seq data in K562 cells revealing interactions between 28S and *SNORD2*, *SNORD18A*, *SNORD18C*, *SNORD26*, *SNORD32A*, *SNORD50A*, and *SNORD60*, respectively. Regions colored in green denote the identified interaction regions. The dashed lines denote previously known 2'-OMe modification sites. KARR-seq was performed in two biological replicates. (**f**) Validation of newly-identified snoRNA-18S interactions by 18S pull-down followed by qPCR in K562 cells. The experiment was performed using three biological samples. Data were shown as mean ± s.d. and were normalized to the level of snoRNAs in the corresponding input samples. *P* values were calculated using two-tailed Student's t-test.

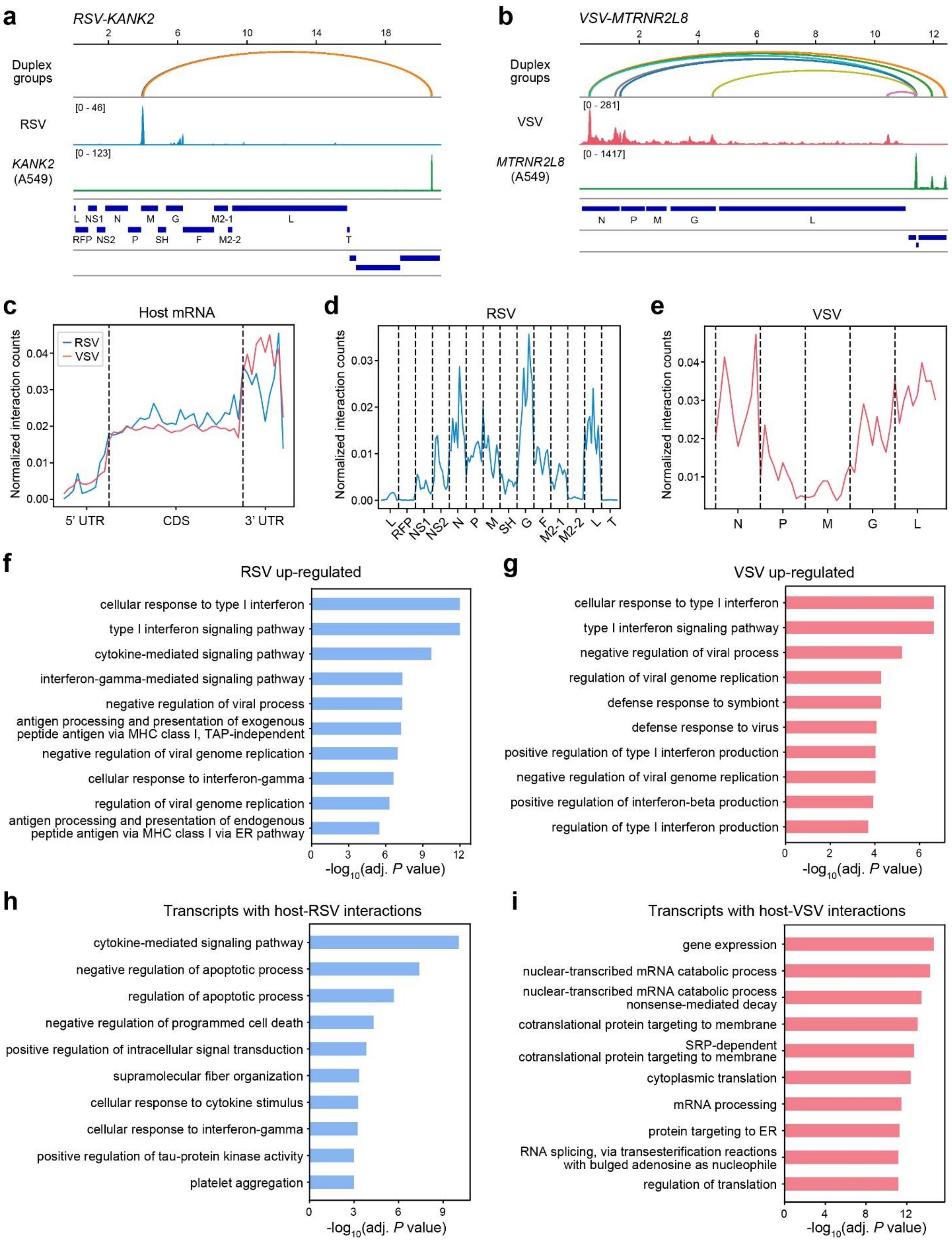

Supplementary Figure 14. RNA–RNA interactions between RSV/VSV and host RNAs. (**a**)–(**b**) Snapshots of KARR-seq data corresponding to *KANK2*–RSV interactions (a) and *MTRNR2L8*–VSV

interactions (b) in A549 cells. (**c**) The relative abundance of KARR-seq chimeric reads that correspond to virus-host RNA–RNA interactions across host mRNA. (**d**)–(**e**) The relative abundance of KARR-seq chimeric reads that correspond to virus-host RNA–RNA interactions across RSV (d) and VSV (e) RNAs. (**f**)–(**g**) Molecular pathways enriched in the up-regulated genes of A549 cells upon RSV (f) and VSV (g) infections. (**h**)–(**i**) Molecular pathways enriched in A549 mRNAs that interact with the RSV (h) and VSV (i) RNAs. KARR-seq was performed in two biological replicates. n=238 transcripts in (f), n=330 transcripts in (g), n=145 transcripts in (h), n=846 transcripts in (i).

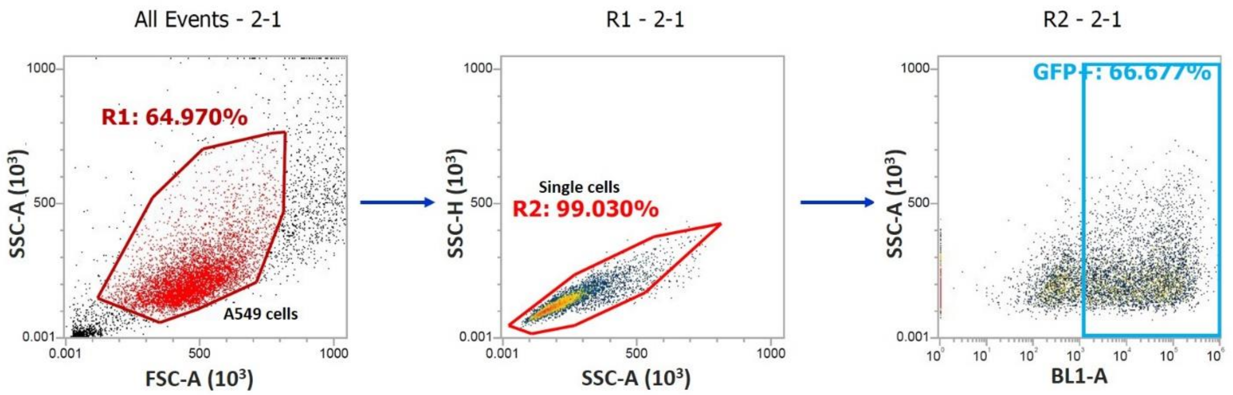

Supplementary Figure 15. Gating strategy to quantify GFP-positive A549 cells. This is used for data presented in Fig. 5 (h) and (i).

Supplementary Fig. 7a

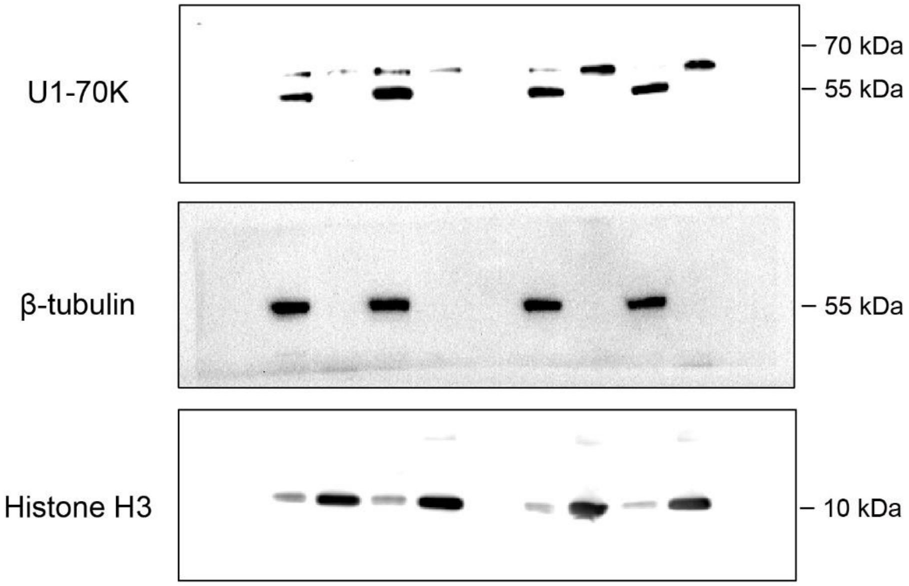

Supplementary Fig. 11d

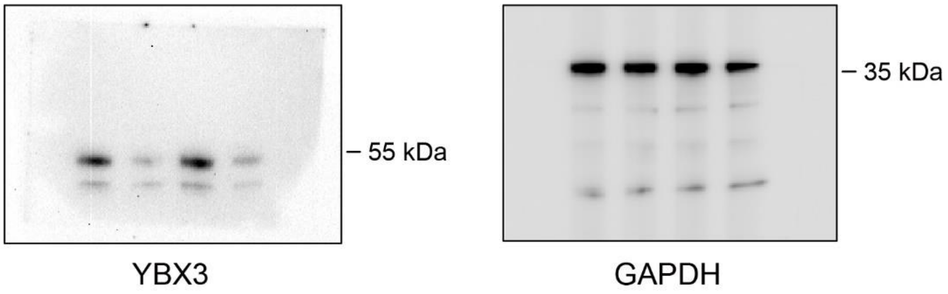

Supplementary Fig. 11e

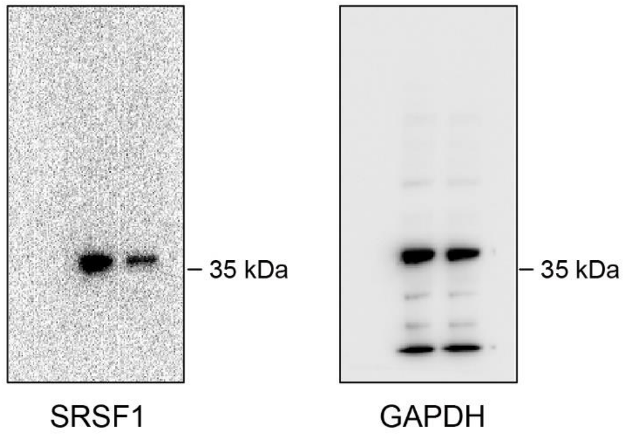

Supplementary Figure 16. Uncropped scans for Western blots.

Supplementary Protocol

KARR-seq protocol using formaldehyde-fixed mammalian cells

I. Cell Fixation

1. Fix cells by suspending cells in their culturing media with a density of 1×10^6 cells/mL. Add formaldehyde (Thermo Fisher, 28906) to a final concentration of 1% (w/v).

2. Rotate the mixture at room temperature for 10 min, then add glycine (from a 500 mM stock solution, homemade by using Sigma-Aldrich, G7126) to a final concentration of 25 mM. Rotate the mixture at room temperature for another 5 min.

3. Centrifuge cells at 1,000 ×g to collect the cell pellet.

II. N_3-kethoxal labeling and crosslinking

4. Use 2-5 million cells for each sample. Resuspend cells into 500 μL permeabilization buffer (10 mM Tris-HCl pH 8.0, 10 mM NaCl, 0.2% IGEPAL CA-630, 5 mM EDTA) supplemented with 2 mM N_3-kethoxal, proteinase inhibitor (Sigma-Aldrich, 11873580011), and SUPERNase inhibitor (Thermo Fisher, AM2696).

5. Rotate the mixture at room temperature for 30 min. Then centrifuge the tube at 2,500 ×g for 5 min.

6. Wash the pellet by resuspending it in 500 μL permeabilization buffer, then centrifuge at 2,500 ×g for 5 min at 4 ℃ to collect the cell pellet. Repeat the wash.

7. Resuspend the pellet in 500 μL lysis buffer supplemented with 12.5 μM G1-DBCO-biotin dendrimer. Shake the mixture at 1,000 rpm at 37 ℃ for 1 h.

8. Centrifuge at 2,500 ×g for 5 min at 4 ℃ to collect cells. Then wash the pellet by resuspending it in 500 μL permeabilization buffer. Collect the cells by centrifuging at 2,500 ×g for 5 min at 4 ℃.

9. Resuspend the pellet in 410 μL 25 mM K_3BO_3. Add 50 μL 10% SDS, 30 μL proteinase K (Thermo Fisher, 25530049), and 10 μL SUPERNase inhibitor. Shake at 55 ℃ for 2 h.

10. Use 500 μL phenol-chloroform (Thermo Fisher, AM9722) to extract RNA from the mixture. Collect the aqueous phase and add 1 mL ethanol, 50 μL 3 M sodium acetate (Thermo Fisher, AM9740), and 1 μL GlycoBlue (Thermo Fisher, AM9515) to precipitate the RNA. Store the mixture at -80 ℃ for at least 4 h or overnight.

III. DNase and proteinase digestion

11. Collect the RNA pellet by centrifuging the mixture at 14,000×g for 15 min at 4 ℃. Then add 500 µL 80% ethanol to wash the pellet and then centrifuge the tube at 14,000×g for 5 min at 4 ˚C.

12. Collect the pellet. Dissolve RNA in 105 µL 25 mM K_3BO_3. Add 12 µL 10× DNase I buffer (Thermo Fisher, AM8170G), 2 µL DNase I (Thermo Fisher, 18047019), and 1 µL SUPERNase inhibitor. Shake at 500 rpm at 37 ℃ for 30 min. Then add 130 µL 2× proteinase K buffer (100 mM Tris-HCl pH 7.5, 200 mM NaCl, 2 mM EDTA, 1% SDS), and 10 µL proteinase K (Thermo Fisher, 25530049). Shake the tube at 500 rpm at 55 ℃ for 30 min.

13. Extract and precipitate RNA by using 250 µL phenol-chloroform following step 10. Add 500 µL ethanol, 25 µL 3 M sodium acetate (Thermo Fisher, AM9740), and 1 µL GlycoBlue (Thermo Fisher, AM9515) to the aqueous phase and precipitate the RNA.

IV. Capture the crosslinked RNA

14. Collect RNA following step 11. Dissolve RNA by adding 63 µL 25 mM K_3BO_3. Add 7 µL 10× RNA fragmentation buffer (Thermo Fisher, AM8740). Incubate the mixture at 70 ℃ for 15 min, then add 8 µL fragmentation stop buffer (Thermo Fisher, AM8740) and put the mixture on ice immediately.

15. Prepare 30 µL Dynabeads Myone Streptavidin C1 (Thermo, 65001) by washing once with 100 µL 1× binding and wash buffer (5 mM Tris-HCl pH 7.4, 0.5 mM EDTA, 1 M NaCl, 0.05% Tween-20), once with 100 µL buffer A (100 mM NaOH, 50 mM NaCl), once with 100 µL buffer B (100 mM NaCl). Then block the beads with 100 µL binding and wash buffer containing 1 µg/µL BSA (NEB, B9000S) and 1 µg/µL salmon sperm DNA (Thermo Fisher, 15632011) by rotating at room temperature for 30 min.

16. Wash beads once with 100 µL 1× binding and wash buffer. Then resuspend beads in 80 µL 2× binding and wash buffer (10 mM Tris-HCl pH 7.4, 1 mM EDTA, 2 M NaCl, 0.1% Tween-20). Mix the beads with the reaction mixture from step 14. Rotate the mixture at room temperature for 20 min.

V. End repair and proximity ligation

17. Collect the beads. Wash the beads twice with 100 µL 1× binding and wash buffer, once with 100 µL 1× PNK buffer (diluted from 10× PNK buffer from NEB, M0201L). Resuspend the beads in 41 µL 25 mM K_3BO_3. Add 5 µL 10× T4 PNK buffer, 3 µL T4 PNK, 1 µL SUPERNase inhibitor. Shake the beads at 1,000 rpm at 37 ℃ for 30 min.

18. Add another 1 µL 10× T4 PNK buffer, 3 µL T4 PNK (NEB, M0201L), and 6 µL 10 mM ATP into the reaction mixture. Shake the tube at 37 ℃ for another 30 min.

19. Wash the beads twice with 100 µL 1× binding and wash buffer and once with 100 µL 1× ligation buffer (diluted from 10× T4 RNA ligase buffer from NEB, M0437M).

20. Resuspend beads in 673 µL 25 mM K_3BO_3, add 100 µL 10× T4 RNA ligase buffer, 2 µL 10 mM ATP, 200 µL 50% PEG8000, 20 µL T4 RNA ligase I (NEB, M0437M), and 5 µL SUPERNase inhibitor. Shake the reaction mixture at 1,000 rpm at 16 ℃ overnight (for around 16 h).

VI. Purification of the ligation product, library preparation, and sequencing

21. Collect beads and wash beads 3 times with 1× binding and wash buffer. Elute RNA by heating the beads in 50 µL H_2O at 95 ℃ for 10 min.

22. Purify the RNA by using the RNA Clean & Concentrator kit (Zymo Research, R1014) following the manufacturer's protocol. Elute RNA with 20 µL H_2O.

23. Measure the concentration of the RNA. Take 10 ng RNA for library construction by using SMARTer Stranded Total RNA-seq kit v2 – pico input mammalian (Takara, 634413).

24. Sequence the library using Illumina platforms using the pair-ended 150 bp mode, aiming for at least 60 million reads per sample.
